# Improvement Effects of Myelophil on Symptoms of Chronic Fatigue Syndrome in a Reserpine-Induced Mouse Model

**DOI:** 10.3390/ijms221910199

**Published:** 2021-09-22

**Authors:** Ji-Hye Song, Seul-Ki Won, Geun-Hyang Eom, Da-Som Lee, Byung-Jin Park, Jin-Seok Lee, Chang-Gue Son, Ji-Yeun Park

**Affiliations:** 1 Department of Korean Medicine, College of Korean Medicine, Daejeon University, Daejeon 34520, Korea; createjh@naver.com (J.-H.S.); wonsulki79@hanmail.net (S.-K.W.); eghss0418@naver.com (G.-H.E.); lls45@naver.com (D.-S.L.); fkao163@gmail.com (B.-J.P.); neptune@dju.kr (J.-S.L.); ckson@dju.kr (C.-G.S.); 2 Institute of Bioscience & Integrative Medicine, Daejeon University, Daejeon 34520, Korea

**Keywords:** chronic fatigue syndrome, myalgic encephalomyelitis, myelophil, herbal medicine, depression, pain, reserpine, TGF-β, serotonin, dopamine, inflammation

## Abstract

Myalgic encephalomyelitis/chronic fatigue syndrome (ME/CFS) is associated with various symptoms, such as depression, pain, and fatigue. To date, the pathological mechanisms and therapeutics remain uncertain. The purpose of this study was to investigate the effect of myelophil (MYP), composed of *Astragali Radix* and *Salviae* *miltiorrhizae Radix*, on depression, pain, and fatigue behaviors and its underlying mechanisms. Reserpine (2 mg/kg for 10 days, intraperitoneally) induced depression, pain, and fatigue behaviors in mice. MYP treatment (100 mg/kg for 10 days, intragastrically) significantly improved depression behaviors, mechanical and thermal hypersensitivity, and fatigue behavior. MYP treatment regulated the expression of c-Fos, 5-HT1A/B receptors, and transforming growth factor β (TGF-β) in the brain, especially in the motor cortex, hippocampus, and nucleus of the solitary tract. MYP treatment decreased ionized calcium binding adapter molecule 1 (Iba1) expression in the hippocampus and increased tyrosine hydroxylase (TH) expression and the levels of dopamine and serotonin in the striatum. MYP treatment altered inflammatory and anti-oxidative-related mRNA expression in the spleen and liver. In conclusion, MYP was effective in recovering major symptoms of ME/CFS and was associated with the regulation of dopaminergic and serotonergic pathways and TGF-β expression in the brain, as well as anti-inflammatory and anti-oxidant mechanisms in internal organs.

## 1. Introduction

Myalgic encephalomyelitis/chronic fatigue syndrome (ME/CFS) is a persistent state of helplessness due to overwork or mental illness that severely deteriorates physical, mental, and occupational quality, leading to social isolation [1]. The most common symptom of ME/CFS is extreme tiredness, which is accompanied by various symptoms, such as impaired memory or concentration, sore throat, tender cervical or axillary lymph nodes, muscle pain, multi-joint pain, headaches, unrefreshing sleep, depression, and post-exertion malaise [2]. ME/CFS is very similar to the representative symptoms of depression and fibromyalgia, but they are distinguished from each other [3]. 

ME/CFS patients have several unique traits compared to depression and fibromyalgia, including decreased serotonin (5-hydroxytryptamine, 5-HT) levels due to upregulation of the 5-HT transporter in astrocytes, which are reported to be related to autoimmune activity via an immune-inflammatory pathway [4]. Previous studies have reported that both oxidative stress and inflammatory cytokine levels were significantly altered in ME/CFS patients [5]. 

Currently, various animal models using drug administration, such as reserpine, poly I:C, lipopolysaccharide, or fluoxetine, and long-period behavioral stress, such as water immersion stress, forced wheel running, or repeated forced swimming, have been utilized for ME/CFS-related studies [6,7,8,9]. Among them, reserpine has been reported to contribute to the reduction of noradrenaline concentration via the regulation of vesicular monoamine transporter2 (VMAT2) in the synaptic cleft [10]. The reserpine-induced rodent model represents depression- and pain-like behaviors, and these behavioral changes are mainly associated with monoaminergic dysfunction related to dopamine and 5-HT uptake suppression [11]. It has also been reported that reserpine treatment induces oxidative stress and neuroinflammation [12]. Thus, reserpine-induced animal models can be used as explanatory models of ME/CFS, specifically targeting depression-, pain-, and fatigue-like behaviors and the associated pathological mechanisms [13,14].

MYP consists of extracts from *Astragali Radix* and *Salviae* *miltiorrhizae Radix* in equal proportions, and includes four active ingredients: astragaloside IV and formononetin in *Astragali Radix* and salvianolic acid B and rosmarinic acid in *Salviae miltiorrhizae Radix* [15]. MYP has been known to ameliorate depression, pain, and fatigue-related symptoms in chronic fatigue-associated patients [16]. MYP regulates the central nervous system via hypothalamus-pituitary-adrenal (HPA) axis activation in uncontrolled disorders induced by emotional stress [17], and reduces elevated oxidative stress and proinflammatory cytokine levels in the brain and other internal organs in a restraint stress-induced mouse model [15]. However, the exact therapeutic mechanisms of MYP in ME/CFS remain unclear. 

In this study, we investigated the effects of MYP treatment on the major symptoms of ME/CFS, such as fatigue, depression, and pain behaviors, in a reserpine-induced mouse model. We also demonstrated the mechanisms of MYP treatment through changes in neurotransmitters and the activation of transforming growth factor beta (TGF-β) and ionized calcium-binding adapter molecule 1 (Iba1) in the brain. Additionally, we explored the anti-inflammatory and antioxidant mechanisms in internal organs.

## 2. Results

### 2.1. Improvement Effect of MYP Treatment on Depression-Like Behaviors

**Figure 1:**
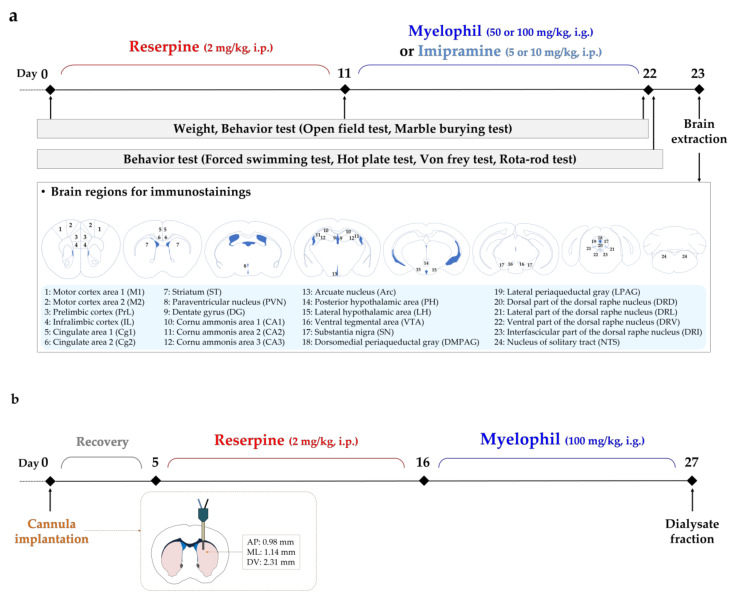
Experimental schedules for behavioral tests and study of brain neuronal mechanisms. (**a**) Schedule for drug administration of reserpine (2 mg/kg, i.p., 10 days), imipramine (5 or 10 mg/kg, i.p., 10 days), and myelophil (MYP: 50 or 100 mg/kg, i.g., 10 days); behavior tests for depression, pain, and fatigue symptoms; and brain extraction for immunostaining in reserpine-induced mice. (**b**) Schedule for microdialysis for conducting neurotransmitters. The cannula was implanted at the striatum (AP: 0.98, ML: 1.14, DV: 2.31 mm). After reserpine and MYP administration, dialysates were collected.

The improvement effect of MYP on depression-like behaviors was demonstrated using the forced swimming test (FST), marble burying test (MBT), and open field test (OFT) on days 0, 11, and 22. After reserpine treatment, MYP was administered at two concentrations of 50 mg/kg (RES + MYP50 group) and 100 mg/kg (RES + MYP100 group); imipramine (IMI), a positive control, was also administered at two concentrations of 5 mg/kg (RES + IMI5 group) and 10 mg/kg (RES + IMI10 group) (Figure 1a).

**Figure 2:**
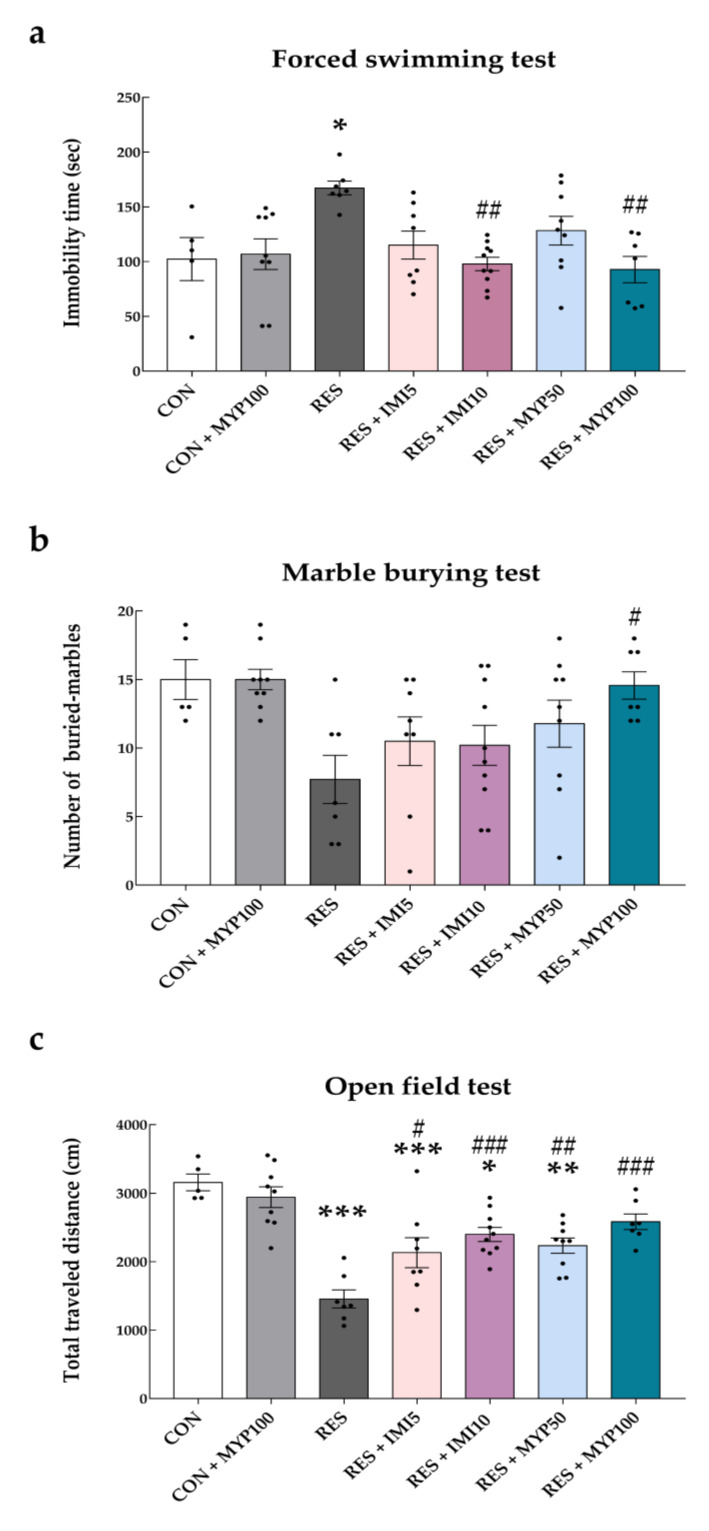
Improvement effects of myelophil (MYP) treatment on depression-like behaviors. Depression-like behaviors were evaluated using the forced swimming test (**a**), marble burying test (**b**), and open-field test (**c**) on day 22. High concentrations of MYP treatment significantly improved depression behaviors. CON: control (*n* = 5), CON + MYP100: 100 mg/kg MYP treatment (*n* = 9), RES: 2 mg/kg reserpine treatment (n = 7), RES + IMI5: 2 mg/kg reserpine and 5 mg/kg imipramine treatment (*n* = 8), RES + IMI10: 2 mg/kg reserpine and 10 mg/kg imipramine treatment (*n* = 10), RES + MYP50: 2 mg/kg reserpine and 50 mg/kg MYP treatment (*n* = 9), RES + MYP100: 2 mg/kg reserpine and 100 mg/kg MYP treatment (*n* = 7). Data are expressed as the mean ± SEM. One-way ANOVA with Tukey post hoc tests was executed. * *p* < 0.05, ** *p* < 0.01, *** *p* < 0.001 vs. CON; ^#^ *p* < 0.05, ^##^ *p* < 0.01, ^###^ *p* < 0.001 vs. RES.

In the FST, the immobility time of mice was significantly higher in the reserpine-treated (RES) group than in the control (CON) group (*p* < 0.05). The RES + IMI10 (*p* < 0.01) and RES + MYP100 (*p* < 0.01) groups showed decreased immobility time compared to the RES group (Figure 2a).

The number of buried marbles was significantly reduced on day 11 in all reserpine-injected groups compared to the CON group (RES: *p* < 0.01, RES + IMI5: *p* < 0.05, RES + IMI10: *p* < 0.001, RES + MYP50: *p* < 0.05, and RES + MYP100: *p* < 0.01 vs. CON group) (Supplementary Figure S1), and it was restored on day 22 only in the RES + MYP100 group (*p* < 0.05 vs. RES group) (Figure 2b).

In the OFT test, the total distance traveled significantly decreased on day 11 in all reserpine-injected groups compared to the CON group (*p* < 0.001) (Supplementary Figure S2). However, it was substantially increased by MYP and IMI treatment in a dose-dependent manner compared to the RES group on day 22 (RES + IMI5: *p* < 0.05, RES + IMI10: *p* < 0.001, RES + MYP50: *p* < 0.01, and RES + MYP100: *p* < 0.001 vs. RES group) (Figure 2c). The zone transition numbers in a restricted space showed a similar tendency, but the differences were not significant (Supplementary Figure S3). 

The MYP-only treatment in control mice (CON + MYP100) had no effect on any of the depression-like behaviors (Figure 2). There was no significant change in the body weight of the mice during the experimental period (Supplementary Figure S4).

According to the results, a high dose of MYP (100 mg/kg) treatment showed the most significant improvement in depression-like behaviors, and the effects of MYP were similar to those of the positive control treatment.

### 2.2. Alteration of Mechanical and Thermal Hypersensitivity by MYP Treatment

**Figure 3:**
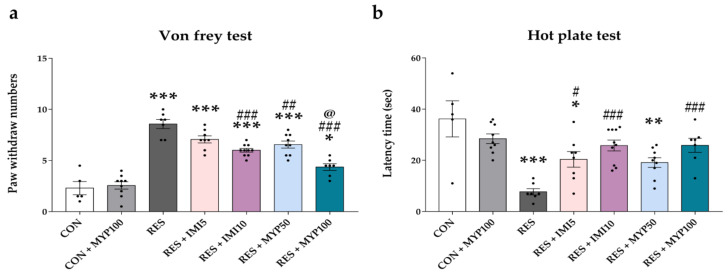
Analgesic effect of myelophil (MYP) treatment on mechanical and thermal hypersensitivity. The von Frey test for mechanical hypersensitivity (**a**) and hot plate test for thermal hypersensitivity (**b**) were performed after MYP treatment on day 22. The latency time and paw withdrawal numbers were significantly improved by imipramine (IMI) and MYP treatment. CON: control (*n* = 5), CON + MYP100: 100 mg/kg MYP treatment (*n* = 9), RES: 2 mg/kg reserpine treatment (*n* = 7), RES + IMI5: 2 mg/kg reserpine and 5 mg/kg imipramine treatment (*n* = 8), RES + IMI10: 2 mg/kg reserpine and 10 mg/kg imipramine treatment (*n* = 10), RES + MYP50: 2 mg/kg reserpine and 50 mg/kg MYP treatment (*n* = 9), RES + MYP100: 2 mg/kg reserpine and 100 mg/kg MYP treatment (*n* = 7). Data are expressed as the mean ± SEM. One-way ANOVA with Tukey post hoc tests was executed. * *p* < 0.05, ** *p* < 0.01, *** *p* < 0.001 vs. CON; ^#^ *p* < 0.05, ^##^ *p* < 0.01, ^###^ *p* < 0.001 vs. RES; ^@^ *p* < 0.05 vs. RES + IMI10.

We performed the von Frey test and hot plate test to measure the mechanical and thermal hypersensitivity of mice on day 22. The paw withdrawal numbers by von Frey filament stimulation for determining mechanical hypersensitivity were significantly increased in the RES group compared to the CON group (*p* < 0.001), and it was diminished after the MYP and IMI treatment (RES + IMI10: *p* < 0.001, RES + MYP50: *p* < 0.01, and RES + MYP100: *p* < 0.001 vs. RES). Among them, the RES + MYP100 group showed the most significant analgesic effects, and the effects were also more powerful than those in the RES + IMI10 group (*p* < 0.05) (Figure 3a). 

The stayed-latency time above the hot plate for measuring thermal hypersensitivity was significantly reduced in the RES group compared to the CON group (*p* < 0.001), but it was significantly increased after the MYP and IMI treatment (RES + IMI5: *p* < 0.05, RES + IMI10: *p* < 0.001, and RES + MYP100: *p* < 0.001) compared to the RES group (Figure 3b). The CON + MYP100 group exhibited no effect on mechanical and thermal hypersensitivity compared to the CON group (Figure 3). 

These results indicate that the analgesic effects of high concentrations of MYP are as effective as the high concentration of the positive control treatment.

### 2.3. Improvement Effect of MYP Treatment on Fatigue Behavior

**Figure 4:**
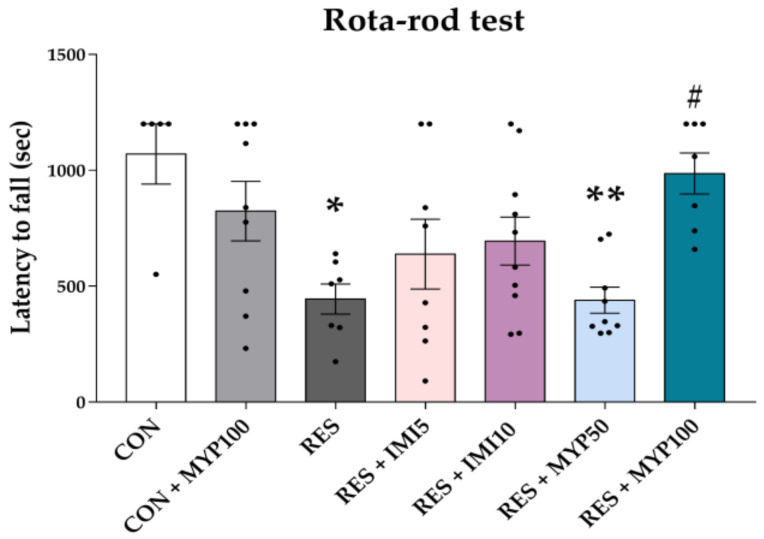
Improvement effect of myelophil (MYP) treatment on fatigue behavior. Rota-rod tests were performed to analyze the fatigue behavior on day 22. The latency time to fall from the rod significantly increased after treatment with 100 mg/kg of MYP. CON: control (*n* = 5), CON + MYP100: 100 mg/kg MYP treatment (*n* = 9), RES: 2 mg/kg reserpine treatment (*n* = 7), RES + IMI5: 2 mg/kg reserpine and 5 mg/kg imipramine treatment (*n* = 8), RES + IMI10: 2 mg/kg reserpine and 10 mg/kg imipramine treatment (*n* = 10), RES + MYP50: 2 mg/kg reserpine and 50 mg/kg MYP treatment (*n* = 9), RES + MYP100: 2 mg/kg reserpine and 100 mg/kg MYP treatment (*n* = 7). Data are expressed as the mean ± SEM. One-way ANOVA with Tukey post hoc tests was executed. * *p* < 0.05, ** *p* < 0.01 vs. CON; ^#^ *p* < 0.05 vs. RES.

The fatigue-like behavior was estimated using the rota-rod test on day 22. The latency time to fall from the rod was decreased in the RES group (*p* < 0.05) compared to the CON group, and it was altered only in the RES + MYP100 group (*p* < 0.05, vs. RES group) (Figure 4).

### 2.4. Changes of c-Fos Expression by MYP Treatment in the Brain

**Figure 5:**
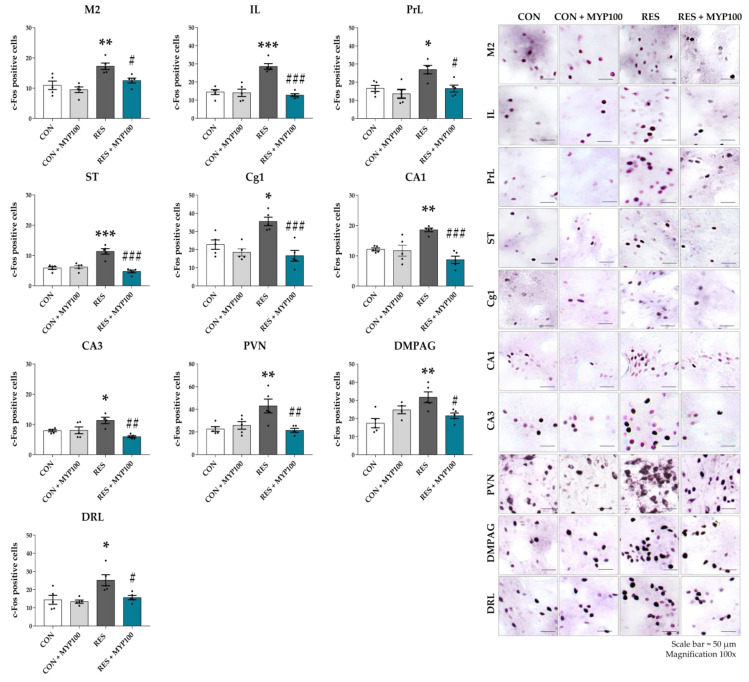
Alteration of c-Fos expression by myelophil (MYP) treatment in each brain regions. The number of c-Fos positive cells in the motor cortex area 2 (M2), infralimbic cortex (IL), prelimbic cortex (PrL), striatum (ST), cingulate area 1 (Cg1), cornu ammonis area (CA) 1 and CA3 of hippocampus, paraventricular nucleus (PVN), dorsomedial periaqueductal gray (DMPAG), and lateral part of the dorsal raphe nucleus (DRL) regions was significantly reduced by 100 mg/kg of MYP treatment. CON: control (*n* = 5), CON + MYP100: 100 mg/kg MYP treatment (*n* = 5), RES: 2 mg/kg of reserpine treatment (*n* = 5), RES + MYP100: 2 mg/kg of reserpine followed by 100 mg/kg of MYP treatment (*n* = 5). Data are expressed as means ± SEM. One-way ANOVA test with Tukey post hoc tests was executed. * *p* < 0.05, ** *p* < 0.01, *** *p* < 0.001 vs. CON; ^#^ *p* < 0.05, ^##^ *p* < 0.01, ^###^ *p* < 0.001 vs. RES.

Expression of c-Fos, a marker of neuronal activity, is commonly induced by inflammatory responses and oxidative stress, as well as by long-term suppression of neurotransmitters due to monoamine depletion [18,19,20]. To identify the pathological condition of the brain tissues by reserpine and the protective effect of MYP, we analyzed the number of c-Fos-positive cells in the 24 brain regions that were highly related to depression, pain, and fatigue behaviors (Figure 1b). The CON + MYP100 group did not exhibit differences in c-Fos expression in the brain compared to the CON group. The number of c-Fos positive cells was increased after reserpine administration in the motor cortex area 2 (M2: *p* < 0.01), infralimbic cortex (IL: *p* < 0.001), prelimbic cortex (PrL: *p* < 0.05), striatum (ST: *p* < 0.001), anterior cingulate area (Cg) 1 (*p* < 0.05), cornu ammonis area (CA) 1 (*p* < 0.01) and CA3 (*p* < 0.05) of the hippocampus, paraventricular nucleus (PVN: *p* < 0.01), dorsomedial periaqueductal gray (DMPAG: *p* < 0.01), and lateral part of the dorsal raphe nucleus (DRL: *p* < 0.05) regions compared to the CON group, and all these changes were altered by MYP treatment (M2: *p* < 0.05, IL: *p* < 0.001, PrL: *p* < 0.05, ST: *p* < 0.001, Cg1: *p* < 0.001, CA1: *p* < 0.001, CA3: *p* < 0.01, PVN: *p* < 0.01, DMPAG: *p* < 0.05, and DRL: *p* < 0.05 vs. RES group) (Figure 5 and Supplementary Figure S5). 

### 2.5. Regulation of 5-HT1A/B Receptors Expression by MYP Treatment in the Brain

Previous studies have reported that the serotonergic pathway is highly related to the major symptoms of ME/CFS, such as fatigue, memory, and depression [21], and the activation of 5-HT1A/B receptors can reflect the release of 5-HT in each brain region [22]**.**

**Figure 6:**
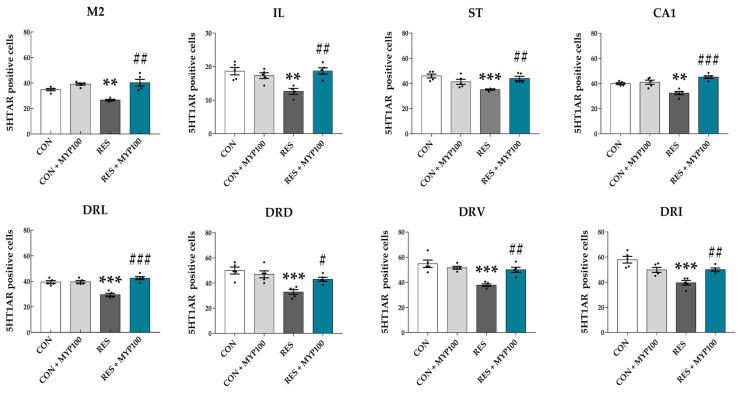
Changes in 5-HT1A receptor (5-HT1AR) expression in each brain region following myelophil (MYP) treatment. The number of 5-HT1AR-positive cells was significantly changed by 100 mg/kg MYP treatment in the motor cortex area 2 (M2), infralimbic cortex (IL), striatum (ST), cornu ammonis area 1 of hippocampus (CA1), lateral part of the dorsal raphe nucleus (DRL), dorsal part of the dorsal raphe nucleus (DRD), ventral part of the dorsal raphe nucleus (DRV), and interfascicular part of the dorsal raphe nucleus (DRI) regions. CON: control (*n* = 5), CON + MYP100: 100 mg/kg MYP treatment (*n* = 5), RES: 2 mg/kg of reserpine treatment (*n* = 5), RES + MYP100: 2 mg/kg of reserpine followed by 100 mg/kg of MYP treatment (*n* = 5). Data are expressed as means ± SEM. One-way ANOVA test with Tukey post hoc tests was executed. ** *p* < 0.01, *** *p* < 0.001, vs. CON; ^#^ *p* < 0.05, ^##^ *p* < 0.01, ^###^ *p* < 0.001 vs. RES.

**Figure 7:**
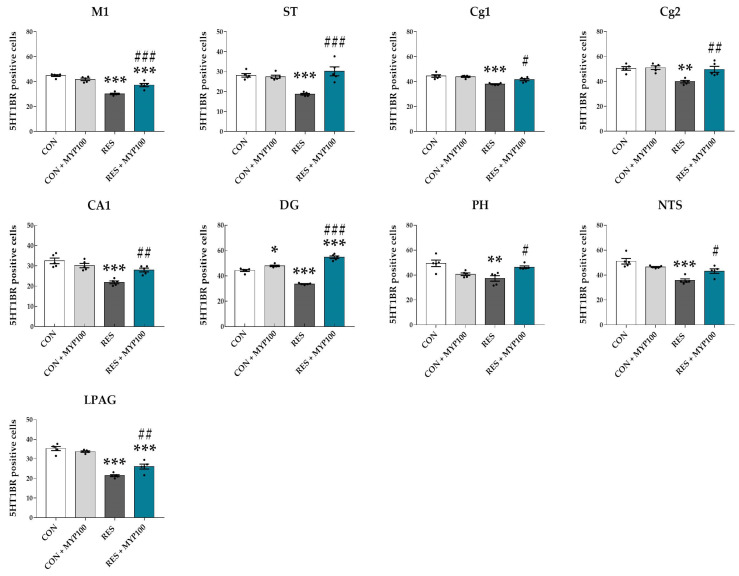
Changes in 5-HT1B receptor (5-HT1BR) expression in each brain region following myelophil (MYP) treatment. The number of 5-HT1BR-positive cells was significantly changed by MYP treatment in the motor cortex area 1 (M1), striatum (ST), cingulate area (Cg) 1, Cg2, cornu ammonis area 1 (CA1) and dentate gyrus (DG) of hippocampus, posterior hypothalamic area (PH), nucleus of solitary tract (NTS), and lateral periaqueductal gray (LPAG) regions. CON: control (*n* = 5), CON + MYP100: 100 mg/kg MYP treatment (*n* = 5), RES: 2 mg/kg of reserpine treatment (*n* = 5), RES + MYP100: 2 mg/kg of reserpine followed by 100 mg/kg of MYP treatment (*n* = 5). Data are expressed as means ± SEM. One-way ANOVA test with Tukey post hoc tests was executed. * *p* < 0.05, ** *p* < 0.01, *** *p* < 0.001 vs. CON; ^#^ *p* < 0.05, ^##^ *p* < 0.01, ^###^ *p* < 0.001 vs. RES.

In the present study, CON + MYP100 group did not exhibit differences in expression of 5-HT1A/B receptors (5-HT1AR and 5-HT1BR) in most brain regions except dentate gyrus (DG: *p* < 0.05 in 5-HR1BR) compared to the CON group (Figure 6 and Figure 7). The number of 5-HT1AR positive cells was significantly decreased in the M2 (*p* < 0.01), IL (*p* < 0.01), ST (*p* < 0.001), CA1 (*p* < 0.01), DRL (*p* < 0.001), dorsal part of the dorsal raphe nucleus (DRD: *p* < 0.001), ventral part of the dorsal raphe nucleus (DRV: *p* < 0.001), and interfascicular part of the dorsal raphe nucleus (DRI: *p* < 0.001) regions after reserpine administration compared to the CON group. All these changes were altered after MYP treatment (M2: *p* < 0.01, IL: *p* < 0.01, ST: *p* < 0.01, CA1: *p* < 0.001, DRL: *p* < 0.001, DRD: *p* < 0.05, DRV: *p* < 0.01, and DRI: *p* < 0.01 vs. RES group) (Figure 6). Similarly, the number of 5-HT1AR positive cells was significantly decreased in the Cg2 (*p* < 0.05), PVN (*p* < 0.001), and lateral hypothalamic area (LH: *p* < 0.01) regions after reserpine administration, but these changes were not altered after MYP treatment (Supplementary Figure S6). 

The number of 5-HT1BR positive cells was significantly decreased in the motor cortex area 1 (M1: *p* < 0.001), ST (*p* < 0.001), Cg1 (*p* < 0.001), Cg2 (*p* < 0.01), CA1 (*p* < 0.001), DG (*p* < 0.001), posterior part of hypothalamus (PH: *p* < 0.01), nucleus of the solitary tract (NTS: *p* < 0.001), and lateral periaqueductal gray (LPAG: *p* < 0.001) regions compared to the CON group. All these changes were altered after MYP treatment (M1: *p* < 0.001, ST: *p* < 0.001, Cg1: *p* < 0.05, Cg2: *p* < 0.01, CA1: *p* < 0.01, DG: *p* < 0.001, PH: *p* < 0.05, NTS: *p* < 0.05, and LPAG *p* < 0.01 vs. RES group) (Figure 7). The number of 5-HT1BR positive cells was also significantly decreased in the PVN (*p* < 0.01), LH (*p* < 0.01), DRL (*p* < 0.05), DRI (*p* < 0.01) and DMPAG (*p* < 0.001) regions, whereas these changes were not altered after MYP treatment (Supplementary Figure S7).

### 2.6. Reduction of TGF-β1 Expression by MYP Treatment in the Brain

**Figure 8:**
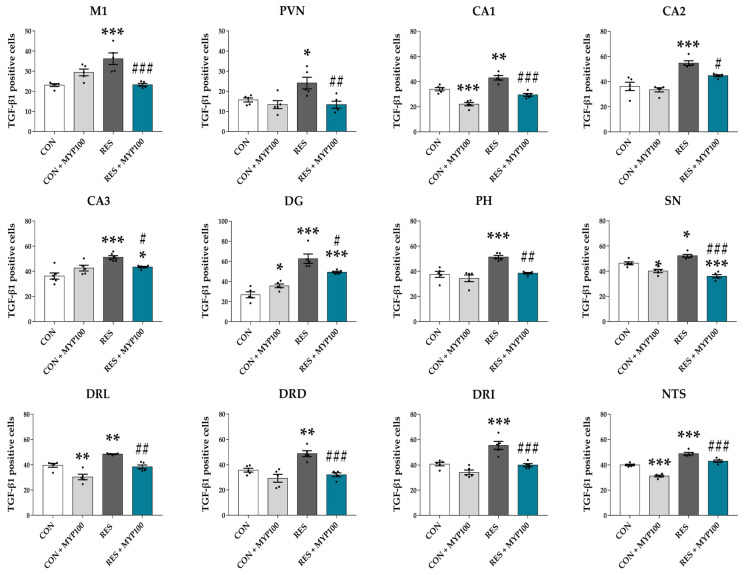
Inhibition of TGF-β expression in each brain region by myelophil (MYP) treatment. The number of TGF-β1 positive cells in the motor cortex area 1 (M1), paraventricular nucleus (PVN), cornu ammonis area (CA) 1, CA2, CA3, and dentate gyrus (DG) of hippocampus, posterior hypothalamic area (PH), substanitia nigra (SN), lateral part of the dorsal raphe nucleus (DRL), dorsal part of the dorsal raphe nucleus (DRD), interfascicular part of the dorsal raphe nucleus (DRI), and nucleus of solitary tract (NTS) regions was significantly decreased by 100 mg/kg MYP treatment. CON: control (*n* = 5), CON + MYP100: 100 mg/kg MYP treatment (*n* = 5), RES: 2 mg/kg of reserpine treatment (*n* = 5), RES + MYP100: 2 mg/kg of reserpine followed by 100 mg/kg of MYP treatment (*n* = 5). Data are expressed as means ± SEM. One-way ANOVA test with Tukey post hoc tests was executed. * *p* < 0.05, ** *p* < 0.01, *** *p* < 0.001 vs. CON; ^#^ *p* < 0.05, ^##^ *p* < 0.01, ^###^ *p* < 0.001 vs. RES.

TGF-β is a multifunctional cytokine involved in immune reactions, cell proliferation, or chemotaxis [23]. Previous studies have reported that TGF-β expression in serum or the brain region is elevated in ME/CFS patients [24]. The CON + MYP100 group exhibited no effect on TGF-β1 expression in most brain regions compared with the CON group, while the number TGF-β1 positive cells was decreased in the CA1 (*p* < 0.001), substantia nigra (SN: *p* < 0.05), DRL (*p* < 0.01), DRV (*p* < 0.05), and NTS (*p* < 0.001) regions and slightly increased in the DG (*p* < 0.05) region compared to the CON group (Figure 8 and Supplementary Figure S8). After reserpine administration, TGF-β1 positive cells were significantly increased in the brain regions M1 (*p* < 0.001), PVN (*p* < 0.05), CA1 (*p* < 0.01), CA2 (*p* < 0.001), CA3 (*p* < 0.001), DG (*p* < 0.001), PH (*p* < 0.001), SN (*p* < 0.05), DRL (*p* < 0.01), DRD (*p* < 0.01), DRI (*p* < 0.001), and NTS (*p* < 0.001) compared to the CON group, and all these changes were significantly altered by MYP treatment (M1: *p* < 0.001, PVN: *p* < 0.01, CA1: *p* < 0.001, CA2: *p* < 0.05, CA3: *p* < 0.05, DG: *p* < 0.05, PH: *p* < 0.01, SN: *p* < 0.001, DRL: *p* < 0.01, DRD: *p* < 0.001, DRI: *p* < 0.001, and NTS: *p* < 0.001 vs. RES group) (Figure 8). The number of TGF-β1 positive cells was significantly decreased in the ST (*p* < 0.001) region after reserpine administration, but this change was not altered by MYP treatment (Supplementary Figure S8).

### 2.7. Analyze Hub Brain Regions According to the Changes of c-Fos, 5-HT1A/B Receptors, and TGF-β1 Expression by MYP Treatment

**Figure 9:**
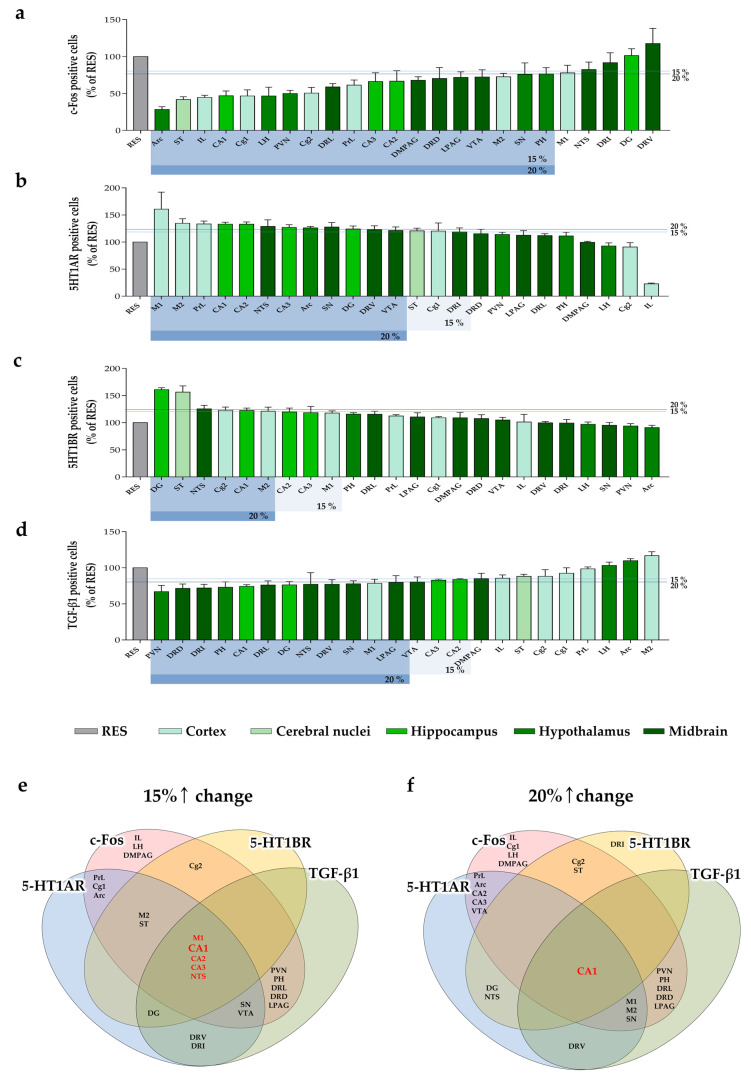
Identification of the major brain regions based on the changes in the expression of c-Fos and 5-HT1A/B receptors and TGF-β1 following myelophil (MYP) treatment. Percentage of cells positive for c-Fos (**a**), 5-HT1A receptor (5-HT1AR) (**b**), 5-HT1B receptor (5-HT1BR) (**c**), and TGF-β1 (**d**) in 24 brain regions after MYP treatment in the order of change. The overlap between the brain regions showing more than 15% (**e**) or 20% (**f**) difference in the expression of c-Fos, 5-HT1A/B receptors and TGF-β1 after MYP treatment.

To identify the most important brain regions involved in the treatment mechanism of MYP, we sorted the changes in c-Fos, 5-HT1AR, 5-HT1BR, and TGF-β1 expression in the RES + MYP100 group in order of change (Figure 9a–d). In addition, the brain regions in which the expression of each factor changed by more than 15% or more than 20% compared to the RES group were selected. The brain regions were then derived, where all changes overlapped. Based on the 15% change, M1, CA1, CA2, CA3, and NTS were derived as brain regions where all factors changed in common (Figure 9e). Furthermore, based on the 20% change, CA1 was derived as the brain region where all factors changed in common (Figure 9f). These results indicate that the motor cortex, hippocampus, and NTS act as key brain regions mediating the therapeutic effect of MYP, and that among these CA1 plays the major role. 

### 2.8. Reduction of TGF-β1 and Iba1 Expression by MYP Treatment in the Hippocampus

**Figure 10:**
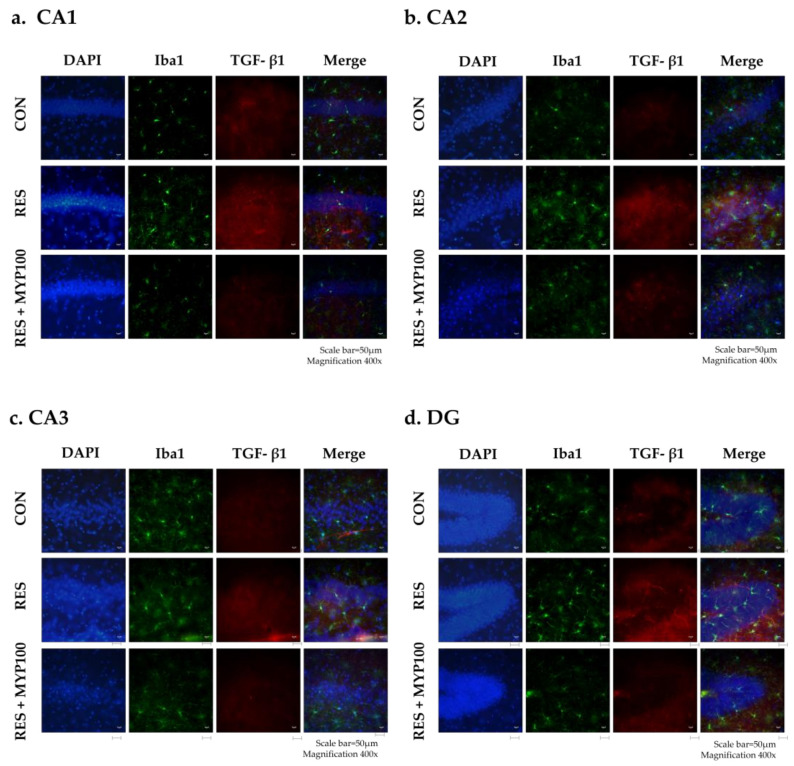
The regulation of TGF-β1 and Iba1 expression by myelophil (MYP) treatment in the hippocampus. (**a**–**d**) DAPI- (blue), TGF-β1- (red), and Iba1- (green) positive cells were observed in cornu ammonis area (CA) 1, CA2, CA3, and dentate gyrus (DG) of the hippocampus. TGF-β1- and Iba1-positive cells were reduced in all hippocampus regions following MYP treatment. Photomicrographs were captured at a magnification of 400× (Scale bar = 50 µm). CON: control, RES: treatment with 2 mg/kg reserpine, RES + MYP100: treatment with 2 mg/kg reserpine followed by 100 mg/kg MYP.

Because hippocampal regions, particularly CA1, were derived as key brain regions mediating the effects of MYP, we identified neuroinflammatory mechanisms in hippocampal regions to investigate further mechanisms. We double stained the TGF-β1 and Iba1 positive cells as neuroinflammatory markers in CA1, CA2, CA3, and DG of the hippocampus. Cells positive for TGF-β1 and Iba1 in all hippocampal areas were prominently increased in the RES group, and they were altered in the RES + MYP100 group (Figure 10).

### 2.9. Effect of MYP Treatment on Dopamine and 5-HT Production in ST

**Figure 11:**
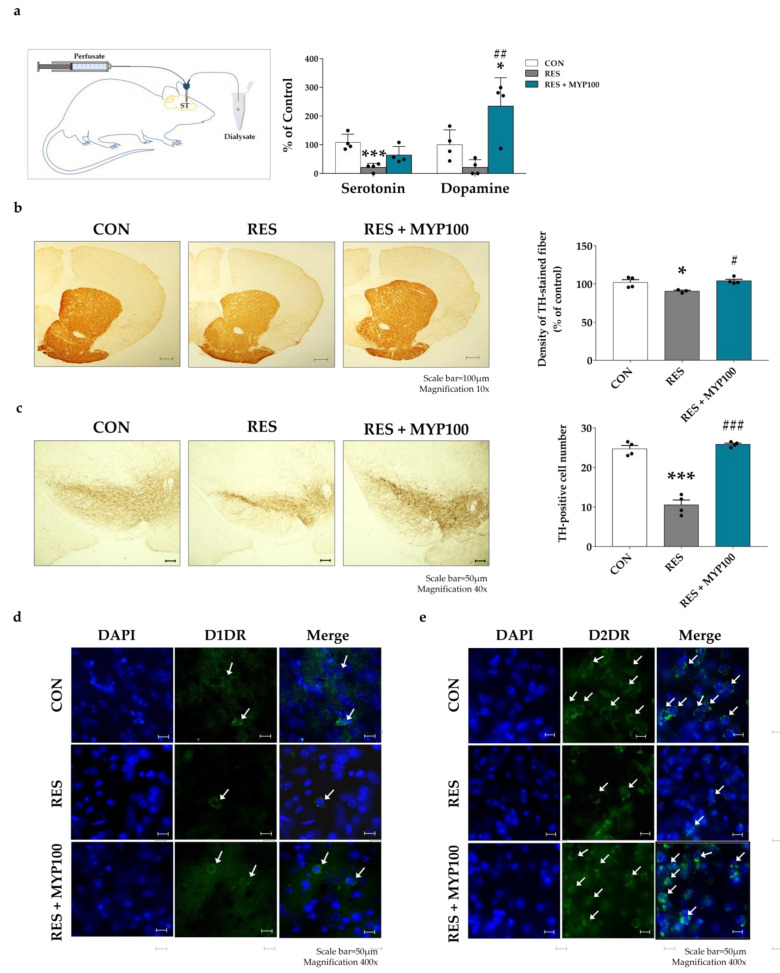
Change in serotonin and dopamine release and the expression of dopamine-related factors in the striatum (ST) following myelophil (MYP) treatment. (**a**) Serotonin and dopamine levels were increased after 100 mg/kg MYP administration on day 27. (**b**) The density of TH-positive fibers in the ST was increased after MYP treatment (Scale bar = 100 µm). (**c**) The number of TH-positive cells in the substantia nigra (SN) region was significantly increased by MYP treatment (Scale bar = 50 µm). (**d**,**e**) Changes in the expression of D1 dopamine receptor (D1DR) and D2 dopamine receptor (D2DR) were observed using immunofluorescence (Scale bar = 50 µm). Expression of D1DR and D2DR increased after MYP treatment. CON: control (*n* = 4), RES: 2 mg/kg of reserpine treatment (*n* = 4), RES + MYP100: 2 mg/kg of reserpine followed by 100 mg/kg of MYP treatment (*n* = 4). Data are expressed as means ± SEM. One-way ANOVA test with Tukey post hoc tests was executed. * *p* < 0.05, *** *p* < 0.001 vs. CON; ^#^ *p* < 0.05, ^##^ *p* < 0.01, ^###^ *p* < 0.001 vs. RES.

The mechanisms of major symptoms of ME/CFS are crucially related to not only the serotonergic pathway but also the dopaminergic pathway [25,26], and ST is the key brain region related to dopaminergic function [26]. We further investigated the release levels of serotonin and dopamine in ST using microdialysis on day 27. The concentration of serotonin in the ST of RES group was significantly lower than that in the CON group (*p* < 0.001). It recovered after MYP treatment, but did not show a significant change. The concentration of dopamine in ST was decreased in the RES group and prominently increased after MYP treatment (*p* < 0.01 vs. RES) (Figure 11a). 

As a noticeable increase in dopamine levels was observed after MYP treatment, we additionally observed dopamine-related factors, such as tyrosine hydroxylase (TH), dopamine receptor D1 (D1DR), and dopamine receptor D2 (D2DR). The density of TH-expressing fiber were significantly decreased in the ST in the RES group compared to the CON group (*p* < 0.05), and it was recovered in the RES + MYP100 group (*p* < 0.05, vs. RES group) (Figure 11b). In addition, the number of TH-positive cells in the SN was significantly reduced in the RES group (*p* < 0.001, vs. CON group), and this effect was reversed after MYP treatment (*p* < 0.001 vs. RES group) (Figure 11c). 

Next, we observed the expression of D1DR and D2DR in ST. The expression of D1DR and D2DR was decreased in the RES group compared to the CON group, and they were increased after MYP treatment (Figure 11d,e). 

These results indicate that the therapeutic effects of MYP treatment are mediated by serotonergic and dopaminergic pathways in the brain. 

### 2.10. Anti-Oxidative and Anti-Inflammation Effects of MYP in Internal Organs

**Figure 12:**
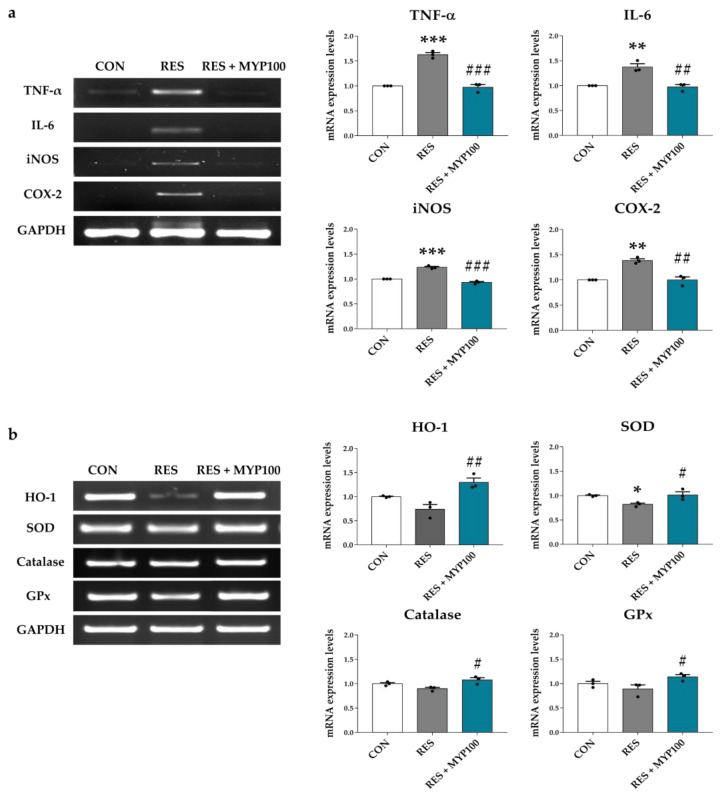
Modulation of anti-inflammation- and anti-oxidation-related genes by myelophil (MYP) treatment in the spleen and liver. (**a**) Expression of anti-inflammation-related mRNAs such as TNF-α, IL-6, iNOS, and COX-2 in spleen was significantly reduced after MYP treatment. (**b**) Anti-oxidation-related mRNAs such as HO-1, SOD, catalase, and GPx in the liver were upregulated after MYP treatment. Gene expression was quantitatively analyzed using Image Lab software. CON: control (*n* = 3), RES: 2 mg/kg of reserpine treatment (*n* = 3), RES + MYP100: 2 mg/kg of reserpine followed by 100 mg/kg of MYP treatment (*n* = 3). Data are expressed as means ± SEM. One-way ANOVA test with Tukey post hoc tests was executed. * *p* < 0.05, ** *p* < 0.01, *** *p* < 0.001 vs. CON; ^#^ *p* < 0.05, ^##^ *p* < 0.01, ^###^ *p* < 0.001 vs. RES.

In the pathological mechanism of ME/CFS, both the brain and internal organs, such as the spleen and liver, play an important role [27]. The increased inflammatory cytokines in the spleen reflect the pathological conditions of ME/CFS patients [28], and liver dysfunction is highly related to fatigue or depression symptoms [29]. We determined the mRNA expression levels of pro-inflammatory cytokines, such as TNF-α, IL-6, iNOS, and COX-2, in the spleen. As shown in Figure 12a, mRNA expression levels of TNF-α, IL-6, iNOS, and COX-2 were significantly increased in the RES group (*p* < 0.001 for TNF-α, *p* < 0.01 for IL-6, *p* < 0.001 for iNOS, and *p* < 0.01 for COX-2 vs. CON group), and were reduced after MYP treatment (*p* < 0.001 for TNF-α, *p* < 0.01 for IL-6, *p* < 0.001 for iNOS, and *p* < 0.01 for COX-2 vs. RES group). 

Then, the effect of MYP treatment on oxidative stress was investigated by measuring antioxidant enzymes such as HO-1, SOD, catalase, and glutathione peroxidase (GPx) mRNA expression levels in the liver. The mRNA expression of HO-1, SOD, catalase, and GPx were decreased in the RES group (*p* < 0.05 in SOD vs. CON group) but increased after MYP treatment (*p* < 0.01 for HO-1, *p* < 0.05 for SOD, *p* < 0.05 for catalase, and *p* < 0.05 for GPx vs. RES group) (Figure 12b).

## 3. Discussion

Our results indicated that MYP improved major symptoms of ME/CFS, such as depression, pain, and fatigue behaviors, in a reserpine-induced mouse model. These therapeutic effects of MYP were associated with the regulation of serotonergic and dopaminergic pathways and TGF-β expression in the brain, as well as the regulation of anti-inflammatory and antioxidant mechanisms in internal organs such as the spleen and liver. 

Previous studies have reported that the prevalence of ME/CFS was estimated to be 0.3–0.6% in the general population [30]. Because the precise pathological mechanisms and therapeutics for ME/CFS are still largely unknown, recent studies have suggested various therapeutics including Western medical drugs, such as antidepressants, physical or mind-body therapy, herbal medicine, and acupuncture. In a clinical study, MYP administration for 3 months in severe ME/CFS patients significantly changed the pain range, results of a questionnaire for evaluating fatigue, and related biomarkers. Lee et al. reported the improvement effect of MYP treatment in depression-like behaviors using FST, OFT, and tail suspension test (TST) in a mouse model of unpredictable chronic mild stress (UCMS), and reported that MYP affected the regulation of 5-HT, TNF-α, and IL-1β expression in the hippocampus and dorsal raphe nucleus (DRN) regions of the brain [31]. Although previous studies have reported that MYP treatment was effective in improving depression-like symptoms, no studies observed all of the major symptoms of ME/CFS, such as depression, pain, and fatigue behaviors. In our results, MYP treatment improved depression, pain, and fatigue behaviors, indicating that MYP treatment could be used to improve the main symptoms of ME/CFS. 

The behavioral symptoms of ME/CFS are closely related to damage to the central nervous system (CNS), particularly the brain. In ME/CFS patients, alterations of neuronal proteins in brain regions occur due to CNS abnormalities caused by the destruction of the HPA axis, changes in serotonergic neurotransmitter systems, and immunological dysfunction [32,33]. According to Akazawa et al., c-Fos expression in the brain regions of a stress-exposed fatigue rat model was sensitively increased in cortical and limbic regions, such as the prefrontal cortex (PFC), lateral septal nucleus, hippocampus, and amygdala [34]. We found that c-Fos expression was specifically increased in several brain regions in a reserpine-induced mouse model, and that the early stages of the nervous system via c-Fos expression induced by reserpine were regulated by MYP. The recovered brain regions by MYP treatment on c-Fos expression were the motor cortex, limbic area, striatum, cingulate cortex, hippocampus, hypothalamus, periaqueductal gray (PAG), and DRN. These regions are strongly related to behavioral changes such as depression, pain, and fatigue, as well as to dopaminergic and serotonergic pathways [35,36,37]. 

Reserpine induces the depletion of monoamine neurotransmitters such as noradrenalin, dopamine, 5-HT, and histamine by blocking vesicular monoamine transporters (VMAT1 and VMAT2) [38]. Levels of 5-HT and dopamine and activation of 5-HT and dopamine receptors are correlated with muscle fatigue or pain during motor activity and depressive and anxiety behaviors [39]. The 5-HT1A/B receptors in the cortex, hippocampus, hypothalamus, DRN, and spinal regions of the serotonergic pathway are involved in mood, emotion, stress responses, and motor activity [40,41]. Previous studies have reported that 5-HT levels are decreased by the upregulation of the 5-HT transporter (5-HTT) in astrocytes in ME/CFS patients [42,43,44,45]. Recently, Lee et al. reported that MYP treatment recovered the altered 5-HT signals in the DRN region in an UCMS animal model [31]. Our results also showed that MYP treatment commonly activated the expression of 5-HT1A/B receptors in ST and CA1, and that the release of 5-HT was restored by MYP treatment in ST. However, since changes in 5-HT release were not directly observed in the hippocampus, more detailed mechanisms need to be elucidated through direct observation using microdialysis in the future.

As can be seen from these results, 5-HT has been studied as a major mechanism of ME/CFS in many studies, but the role of other neurotransmitters has not been elucidated. Although dopamine is known to be a factor involved in fatigue regulation, no studies have directly observed the changes in dopamine release in brain regions in ME/CFS studies using animal models. The ST and SN are key brain regions for dopamine synthesis, and dopaminergic neurons via several dopaminergic pathways are closely connected to various cortical areas [45]. D1DR and D2DR among dopamine receptors were mostly found in the striatum more than the PFC region; they were associated with the CNS and immune system [46]. We further investigated the dopaminergic pathway transition by analyzing the dopamine release level and expression of D1DR and D2DR in the ST region after MYP treatment. The observed increase in dopamine release and increase in dopamine receptor expression in our results suggests that the therapeutic effect of MYP treatment is mediated by regulating serotonergic and dopaminergic pathways. 

Imbalances in the dopamine and 5-HT pathways are also caused by dysfunction of the immune system associated with the release of inflammatory cytokines, such as TNF-α, IL-1, IL-6, and IFN-γ [47]. In addition, TGF-β in cerebrospinal fluid (CSF) in severe exercise-induced fatigue models can induce Alzheimer’s disease by producing amyloid-β and suppressing neural stem cell proliferation [48]. Clark et al. and Montoya et al. reported that only TGF-β levels were significantly elevated in patients with ME/CFS [49,50]. In our results, TGF-β1 levels were mainly increased in the motor cortex, hippocampus, hypothalamus, DRN, and NTS regions of the reserpine-induced mouse model, but they were reduced by MYP treatment. These pro-inflammatory cytokine-stimulated brain regions were similar to those of the serotonergic and dopaminergic pathway-related brain regions. 

Thus, we identified hub brain regions where the expression of c-Fos, 5-HT1A/B receptors, and TGF-β1 were commonly observed. Five brain regions, M1, CA1, CA2, CA3, and NTS were derived as key regions that mediated MYP effects, and among them, CA1 of the hippocampus was the most strongly contributing to the MYP effects. These brain regions are connected with other brain regions that are related to emotional changes, learning, and motor activity, similar to ME/CFS behavior symptoms. Dysfunction of the hippocampus in ME/CFS patients is closely related to symptoms, such as neuroendocrine dysfunction, pain perception, memory impairment, and hypersensitivity to stress. In particular, the CA1 region of the hippocampus is known to be a major region that regulates cognitive impairment through DG and CA3 and controls stress response through indirect pathways from the subiculum to the hypothalamus by numerous limbic nuclei. Alterations of the CA1-connected subiculum are transmitted to the PFC, thalamus, septum, amygdala, and NTS, and produce behavioral symptoms of ME/CFS [51]. Therefore, CA1, which is centrally involved in changes in various biomarkers in the brain, can act as a major brain region mediating the behavioral improvement and therapeutic mechanisms of MYP. 

ME/CFS has been reported as a multisystemic disease in which neuronal function is inhibited by the activation of ROS/RNS and immuno-inflammatory pathways [51]. Increased ROS induced by oxidative stress in the pathological mechanism of ME/CFS induces depression- or pain-like behaviors through decreased levels of anti-oxidative enzymes, such as SOD, catalase, and glutathione, and increased lipid peroxidation [52]. Meanwhile, scavenging of oxygen free radicals by antioxidants induces the activation of antioxidant enzymes and inhibition of cytokine release by nitric oxide synthase (NOS), contributing to the prevention or treatment of ME/CFS [53]. The treatment effects of MYP are known to prevent antioxidant system-related hepatic injury by altering aspartate aminotransferase (AST) and alanine aminotransferase (ALT) levels in a restraint stress-induced animal model [50]. MYP treatment also regulates the oxidative stress, such as ROS, NO, GSH, SOD, and catalase, and inflammatory cytokines, such as TNF-α, IL-1β, IL-6, and IL-10 in skeletal muscles of chronic forced exercise-induced chronic fatigue animal models [31]. Our results demonstrated that inflammatory markers, such as TNF-α, IL-6, iNOS, and COX-2, in the spleen and the oxidative stress-related markers, such as HO-1, SOD, catalase, and GPx, in the liver, were regulated by MYP treatment. These results suggest that the protective effect of MYP may variously contribute to the normalization of the CNS through the regulation of pro-inflammatory cytokines and oxidative stress-related substances in the brain and other organs related to ME/CFS.

This study is significant in that we demonstrated that MYP treatment improved all major symptoms related to ME/CFS, such as depression, pain, and fatigue behavior, and that detailed mechanisms were induced through the brain and intestines. However, the detailed mechanisms of ME/CFS pathogenesis and MYP treatment need to be further investigated. In the future, it will be necessary to study the neurobiological mechanisms through analysis of biologically active substances of MYP components and changes in other neurotransmitters in relation to the improvement of ME/CFS symptoms. In addition, differences in neurophysiological mechanisms by sex should also be investigated in the future.

## 4. Materials and Methods

### 4.1. Animals

Male C57BL/6 mice (6–7 weeks old, 20–25 g) used in this study were obtained from Daehan Biolink (Eum-seong, Chungcheongbuk-do, Korea). All mice were randomly divided and four were placed in each cage. Mice were acclimated for at least 1 week before the experiments with free access to water and food and with a 12-h light/dark cycle at 23 ± 1 °C. All experimental protocols used in this study were approved by the Institutional Animal Care and Use Committee (IACUC) at Daejeon University (approval no. DJUARB2019-037).

### 4.2. Reserpine Injection and MYP Administration

After 1-week of acclimatization, reserpine (2 mg/kg in 0.05% acetic acid, i.p., Sigma; St. Louis, MO, USA) was injected intraperitoneally to mice for 10 days to induce major symptoms of ME/CFS, such as fatigue, depression, and pain behaviors. Then, MYP (50 or 100 mg/kg dissolved in saline; KB-Myelo-1801, Kyung-Bang Pharmacy, Incheon, Republic of Korea) was administered intragastrically (i.g.) for 10 days (from day 12 to day 22). IMI (5 and 10 mg/kg dissolved in saline; Sigma, St. Louis, MO, USA) was administered as a positive control for the same period. A total of 55 mice were randomly assigned to seven groups: (1) CON: control, (2) CON + MYP100: 100 mg/kg of myelophil administration, (3) RES: reserpine administration, (4) RES + IMI5: reserpine and 5 mg/kg of imipramine administration, (5) RES + IMI10: reserpine and 10 mg/kg of imipramine administration, (6) RES + MYP50: reserpine and 50 mg/kg of MYP administration, (7) RES + MYP100: reserpine and 100 mg/kg of MYP administration. For the control treatment, 0.05% acetic acid was injected as a control for reserpine, and saline was administered as a control for MYP or IMI. 

### 4.3. Behavioral Test

After reserpine administration, behavioral analyses were performed to analyze depression, pain, and fatigue behaviors on days 0, 11, and 22. The open field and marble burying tests were performed on days 0, 11, and 22, and the forced swimming, hot plate, von Frey, and rota-rod tests were performed on day 22 (Figure 1a). 

#### 4.3.1. OFT

Mice were stabilized in a test room for more than 1 h prior to the behavioral test. They were placed inside a box (30 × 30 × 30 cm) for 5 min and then the total distance and zone transition number were measured using a video camera system to track movement (SMART 3.0; Panlab S. L., Barcelona, Spain) for 10 min. 

#### 4.3.2. MBT

To evaluate depression-like behavior, the MBT was conducted according to the method described by Deacon et al. [54]. Twenty marbles (1.8 cm in diameter) at regular intervals were placed on a 5-cm high bedding in a clear polypropylene cage (20 × 26 × 13 cm), and each mouse was placed in an individualized cage for 30 min. Then, the number of marbles hidden to a depth of 2/3 in the bedding was counted.

#### 4.3.3. FST

FST was estimated according to the method described by Eckeli et al. [55]. The mice were individually forced to swim in an open cylindrical container (10 cm diameter, 25 cm height) filled with 19 cm of water at 25 ± 1 °C. Mice were adapted to the water for 2 min, and the immobility time was measured for 4 min using a video camera system (SMART 3.0; Panlab S. L., Barcelona, Spain).

#### 4.3.4. Von Frey Test

A von Frey filament (II TC, Woodland Hills, CA, USA) was used to measure the hind paw withdrawal threshold. Prior to the test, all mice were acclimatized in a clear acrylic box (10 × 10 × 10 cm) with a mesh bottom for 20 min, and the surfaces of the bilateral hind paws were stimulated with von Frey filament hair exerting a constant force (1.3 g) at 5 s intervals. The number of licking or quick withdrawing of the hind paw was counted out of a total of 10 stimulations.

#### 4.3.5. Hot Plate Test

The hot plate test was performed according to the method described by Derrien et al. [56]. Mice were placed on a heated metal plate at 52 ± 1 °C, and the latency time and response number of nociceptive reactions, such as licking, waving, or jumping with one of the feet were measured for 60 s.

#### 4.3.6. Rota-Rod Test

All mice were trained for 3 days before the experiment using the rota-rod test (MED Associates Inc., St. Albans, VT, USA). Mice were trained with fixed speed settings for 240 s (20 rpm for 60 s, 24 rpm for 60 s, 28 rpm for 60 s, and 32 rpm for 60 s) for practice. On day 22, the latency time to fall from the rod was measured at the accelerating speed setting (3.5–35 rpm) for 1200 s.

### 4.4. Immunostainings

Mice were anesthetized with 2.5% isoflurane (Pharm Co., Ltd., Hwaseong-si, Gyeonggi-do, Korea) and 100% oxygen mixture using an anesthesia machine (R510IP, RWD Life Science Co., Ltd., China). Mice were transcardially perfused with 0.05 M phosphate-buffered saline (PBS, GeneAll, Sonhpa-gu, Seoul, Korea) buffer, followed by fixation with 10% paraformaldehyde (PFA, Sigma, St. Louis, MO, USA). The brains were then extracted and post-fixed overnight in 4% PFA (Sigma, St. Louis, MO, USA) at 4 °C. The brains were then sunk in a gradient of 10%–30% sucrose solution and cut into coronal sections of 40 μm thickness using a cryostat (CM3050S, Leica Microsystems, Nussloch, Germany). Then, the brain tissues were stored in an anti-freeze storing solution. 

For immunohistochemistry, brain tissues were washed with PBS containing 0.1% Triton x-100 (PBST). Endogenous peroxidase in the brain tissue was inactivated by 3% H₂O₂ for 20 min and then tissues were incubated with 1% bovine serum albumin (BSA, BOVOGEN, Williams Ave, Keilor East VIC, Australia) at room temperature for 1 h. Tissues were incubated with primary antibodies as follows: c-Fos (1:250; Abcam, AB222699, Cambridge, MA, USA), 5-HT1AR (1:100; Novus Biologicals, NBP2-21590, Centennial, CO, USA), 5-HT1BR (1:100; Abcam, AB13896, Cambridge, MA, USA), TGF-β1 (1:100; Abcam, AB215715, Cambridge, MA, USA), and TH (1:100; Abcam, AB112, Cambridge, MA, USA) at 4 °C overnight. They were then incubated with biotinylated secondary antibody, anti-rabbit IgG (H + L) (1:1000 for c-Fos, 5-HT1AR, and 5-HT1BR, and 1:500 for TGF-β1; Vector Laboratories, BA-1000, Burlingame, CA, USA) at room temperature for 1 h. After washing, the tissues were reacted with ABC reagent and DAB kit. After dehydration with a gradient of 70–100% ethyl alcohol and 100% xylene, brain sections were mounted using permount solution. The stained brain sections were captured using a microscope (Nikon, Minato, Japan), and the number of stained cells in each brain region was manually counted within a square of 32 × 32 μm. The mean values for the left and right regions were calculated. All procedures were blindly performed to minimize observer bias, and the counting process was randomly confirmed by an independent researcher. 

For immunofluorescence, brain tissues were washed with PBST, and antigen retrieval was performed using Citrate Plus HIER Solution (ScyTek Laboratories Inc., West Logan, Virginia, USA). Brain tissues were incubated with 1% BSA for 1 h at room temperature and then incubated with primary antibodies against D1DR (1:200; Santa Cruz, sc-33660, Dallas, TX, USA), D2DR (1:200, Santa Cruz, sc-5303, Dallas, TX, USA), and a mixture of Iba1 (1:200, FUJIFILM Wako Pure Chemical Corporation, 019-19741, Osaka, Japan) and TGF-β1 (1:50; Santa Cruz, sc-139348, Dallas, TX, USA) at 4 °C overnight. They were then incubated with goat anti-rabbit IgG secondary antibodies (1:1000 for Iba1; Alexa Fluor 488; Invitrogen, A11034, Waltham, MI, USA), goat anti-mouse IgG (1:500 for TGF-β1; 1:1000 for D1DR and D2DR; Alexa Fluor 488; Invitrogen, A32723, Waltham, MI, USA) at room temperature for 2 h. After washing with PBST, VECTASHIELD mounting medium stained with 4,6-diamidino-2-phenylindole (DAPI; Vector Laboratories, Burlingame, CA, USA) was mounted. Immunoreactive brain sections were observed using a fluorescence microscope (Nikon, Minato, Japan).

### 4.5. Brain Microdialysis

Mice were anesthetized with a 2.5% isoflurane and 100% oxygen mixture using an anesthesia machine (R510IP, RWD Life Science Co., Ltd., China) and mounted in the frame of a stereotaxic instrument (StereoDrive, Neurostar, Tubingen, Germany). A microdialysis guide cannula (CMA7 Guide Cannula, Harvard Apparatus, Holliston, MA, US) was stereotaxically implanted at the following coordinates: anterior–posterior +0.98, lateral 1.14, vertical −2.31 mm from the bregma and the dual surface in the striatum of the mouse brain region, according to the atlas of PAXINOS, G (2019) [57]. The guide cannula was fixed firmly to the skull with the anchor screws using dental cement (Poly-F Plus, DENTSPLY SIRONA, York, PA, USA). Mice were recovered at least 5 days after the surgery, and then they were administered reserpine (2 mg/kg in 0.05% acetic acid, i.p.) or saline for 10 days. MYP was administered for 10 days and CMA 7 microdialysis probes (0.24 mm i.d., molecular weight cut-off 6 kDa, 1 mm membrane length; Harvard Apparatus, Holliston, MA, USA) were inserted into the guide cannulas of the surviving mice (Figure 1b). The dialysates were perfused from the microdialysis probe using CMA perfusion fluid (1 μL/min of flow). They were collected using a CMA 470 refrigerated microfraction collector (Harvard Apparatus, Holliston, MA, USA) and stored at −70 °C. 

### 4.6. Liquid Chromathography (LC)-Tandem Mass Spectrometer (MS/MS) Analysis

Quantitative analysis of neurotransmitters was performed by LC-MS/MS analysis from NruroVIS (Cheoana, Chungcheongnam-do, Korea).

LC was conducted using SCIEX ExionLC^TM^ UHPLC (Applied Biosystems Corporation, Framingham, MA, USA) with an analytical column (ACQUITY UPLC HSS T3, 2.1 × 100 mm, 1.8 μm, Waters, Milford, MA, USA), and the column oven was maintained at 50 °C. The mobile phase was composed of 0.1% formic acid and 5 mM ammonium formate in water (mobile phase A) and 5 mM ammonium formate in ACM/MeOH (1:1) as mobile phase B. During the analysis, the gradient elution was transformed to 5–90% of mobile phase B and the flow rate was sustained 0.3 mL/min. The injection volume was 10 μL, and the total run time was 7 min. Mass spectrometric analysis was performed using a SCIEX Triple Quadrupole 6500+ (Applied Biosystems Corporation, Framingham, MA, USA) with an electrospray ionization source in positive ion mode with the following parameters: curtain gas 30; collision gas medium; ion source gas 1 50; ion source gas 2 60. The positive ion spray voltage was set to 5000 V. The quantitative results of dopamine and serotonin on the dialysate were calculated as a percentage of the control. 

### 4.7. Reverse Transcription-Polymerase Chain Reaction (RT-PCR)

The mRNA expression levels of HO-1, catalase, SOD, and GPx in the liver and IL-6, TNF-α, COX-2, and iNOS in the spleen were detected using RT-PCR. Total RNA from the liver and spleen was extracted using TRIzol reagent (Takara, Kyoto, Japan). Total RNA concentration and purity were determined using a NanoDrop 2000 spectrophotometer (Thermo Scientific, Waltham, MA, USA). The cDNA was reverse-transcribed using total RNA (1 μg) and M-MLV reverse transcriptase (Enzynomics, Yuseong-gu, Daejeon, Republic of Korea) at 42 °C for 1 h, and then PCR was performed using an Amplification Thermal Cycler (Xi’an Tianlong Science and Technology Co. Ltd., Zhuhong Road, Xi’an, China). Primer information and synthetic conditions are described in Supplementary Table S1. The amplified PCR products were electrophoresed on a 1.5% agarose gel and exposed using a ChemiDoc™XRS+ imaging system (Bio-Rad, Richmond, CA, USA).

### 4.8. Statistical Analyses 

All data are expressed as mean ± standard error (SEM). Data normality was assessed by the D’Agostino-Pearson omnibus normality test, and the outlier data was confirmed by the Robust Regression and Outlier Removal (ROUT) method. Then, verified data were analyzed by one-way ANOVA followed by Tukey post hoc tests using GraphPad Prism 7.0 (GraphPad Software Inc., San Diego, CA, USA). Significance was set at *p* < 0.05. 

## 5. Conclusions

Reserpine-induced mice showed signs of severe depression, pain, and fatigue, which are major symptoms of ME/CFS. High doses of MYP treatment improved depression, pain, and fatigue behaviors in reserpine-induced mice, and these therapeutic effects were associated with the regulation of c-Fos, 5-HT1A/B receptors, and TGF-β1 expression in the brain. Among the brain regions, the motor cortex, hippocampus, and NTS are important brain regions that mediate the therapeutic effect of MYP, and the CA1 area of the hippocampus was derived as the most important region. In addition, MYP treatment regulates serotonergic and dopamine pathways in the brain and regulates anti-inflammatory and antioxidant mechanisms in the internal organs, such as the spleen and liver. Further studies are needed regarding the bioactive substance of MYP, distinct causes, and underlying mechanisms for ME/CFS treatment.

## Supplementary Materials

The following are available online at https://www.mdpi.com/article/10.3390/ijms221910199/s1.
