# Supplementary material for: Improvement Effects of Myelophil on Symptoms of Chronic Fatigue Syndrome in a Reserpine-Induced Mouse Model
Source: Int J Mol Sci. 2021 Sep 22;22(19):10199. doi: 10.3390/ijms221910199 (PMC8508381; doi:10.3390/ijms221910199)
Supplement: Supplementary file 1 [file ijms-22-10199-s001.zip › ijms-1343617 - Supplementary - revised.pdf]

Table S1. Primers used for RT-PCR.

Target	Primer sequence	Annealing temperature (℃)	Cycle
TNF-α	F: 5′-TGGGAGGAAAGGGGTCTAAG-3′<br>R: 5′-TAGCAAATCGGCTGACGGTG-3′	60.5	32
IL-6	F: 5′-GTGGCTAAGGACCAAGACC-3′<br>R: 5′-TTTGGGGGAGGAGGATGTTTGGAT-3′	61	33
iNOS	F: 5′-TGAGGCTGAAATCCCAGCAG-3′<br>R: 5′-AGGCCTCCAATCTCTGCCTA-3′	60	33
COX-2	F: 5′-TGAGTACCGCAAACGCTTCT-3′<br>R: 5′-GGACAAACACCGGAGGGAAT-3′	61	33
HO-1	F: 5′-TGACACCTGAGGTCAAGCAC-3′<br>R: 5′-TTGGACAGAGTTCACAGCCC-3′	60.5	32
SOD	F: 5′-GAACCATCGACGGCCTAGAG-3′<br>R: 5′-CCCCTCATCGATAACCAGGC-3′	60.5	32
Catalase	F: 5′-GAGGAAACGCCTGTGTGAGA-3′<br>R: 5′-TCCCTTCAGGAAACGGCATC-3′	60.5	32
GPx	F: 5′-CAGTCCACCGTGTATGCCTT-3′<br>R: 5′-GTAAAGAGCGGGTGAGCCTT-3′	60.5	32
GAPDH	F: 5′-ACCACAGTCCATGCCATCAC-3′<br>R: 5′-ACCACAGTCCATGCCATCAC-3′	60.5	32

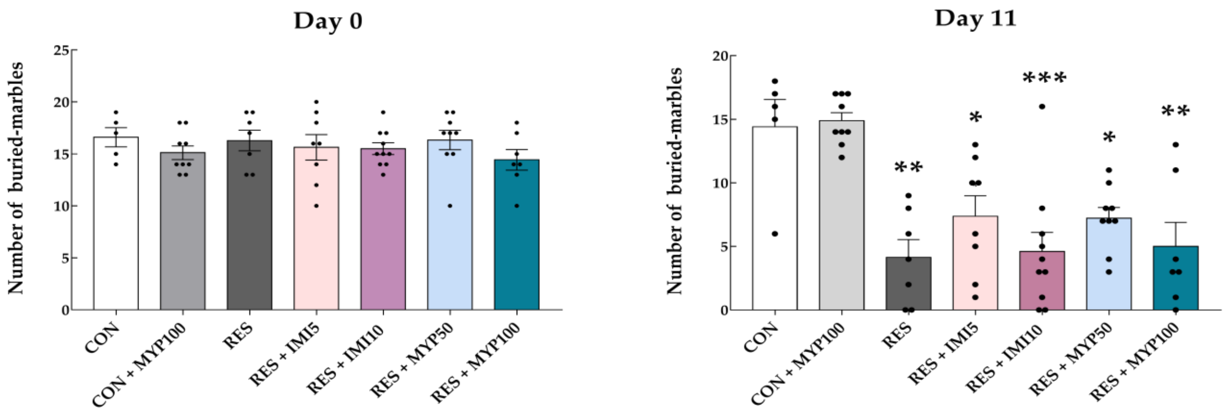

Figure S1. Change in buried-marble number on day 0 (before reserpine treatment) and day 11 (after reserpine treatment). The number of buried marbles was significantly reduced on day 11 in all the reserpine-injected groups. CON: control (n = 5), CON + MYP100: 100 mg/kg MYP treatment (n = 9), RES: 2 mg/kg reserpine treatment (n = 7), RES + IMI5: 2 mg/kg reserpine and 5 mg/kg imipramine treatment (n = 8), RES + IMI10: 2 mg/kg reserpine and 10 mg/kg imipramine treatment (n = 10), RES + MYP50: 2 mg/kg reserpine and 50 mg/kg MYP treatment (n = 9), RES + MYP100: 2 mg/kg reserpine and 100 mg/kg MYP treatment (n = 7). Data are expressed as means ± SEM. One-way ANOVA test with Tukey post-hoc tests executed. $^{*}p < 0.05$, $^{**}p < 0.01$, $^{***}p < 0.001$ vs. CON.

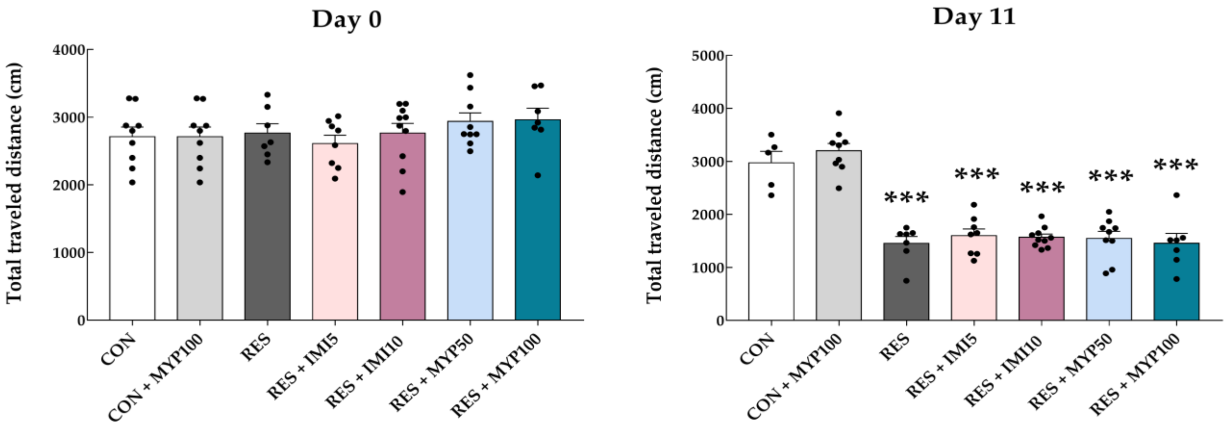

Figure S2. Change in total traveled distance on day 0 (before reserpine treatment) and day 11 (after reserpine treatment). The total distance traveled was determined before (day 0) and after (day 11) reserpine treatment for the evaluation of reserpine-induced depression behavior. CON: control (n = 5), CON + MYP100: 100 mg/kg MYP treatment (n = 9), RES: 2 mg/kg reserpine treatment (n = 7), RES + IMI5: 2 mg/kg reserpine and 5 mg/kg imipramine treatment (n = 8), RES + IMI10: 2 mg/kg reserpine and 10 mg/kg imipramine treatment (n = 10), RES + MYP50: 2 mg/kg reserpine and 50 mg/kg MYP treatment (n = 9), RES + MYP100: 2 mg/kg reserpine and 100 mg/kg MYP treatment (n = 7). Data are expressed as means ± SEM. One-way ANOVA test with Tukey post-hoc tests executed. $^{***}p < 0.001$ vs. CON.

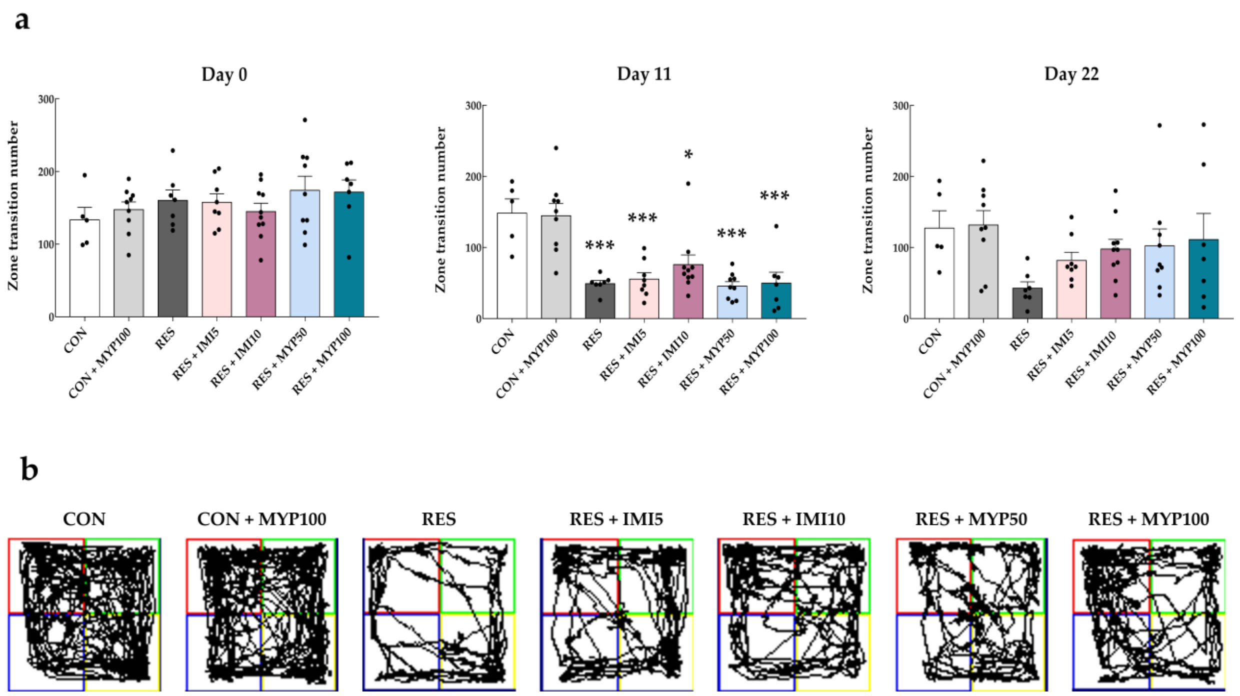

Figure S3. Effect of myelophil (MYP) treatment on zone transition number of open field test on days 0 (before reserpine treatment), 11 (after reserpine treatment), and 22 (after MYP treatment). (a) The zone transition number for the determination of depression behavior was significantly decreased on day 11 in all the reserpine-injected groups, and increased after imipramine (IMI) or MYP treatment on day 22. (b) Thigmotaxis-related images of each groups on day 22. CON: control (n = 5), CON + MYP100: 100 mg/kg MYP treatment (n = 9), RES: 2 mg/kg reserpine treatment (n = 7), RES + IMI5: 2 mg/kg reserpine and 5 mg/kg IMI treatment (n = 8), RES + IMI10: 2 mg/kg reserpine and 10 mg/kg IMI treatment (n = 10), RES + MYP50: 2 mg/kg reserpine and 50 mg/kg MYP treatment (n = 9), RES + MYP100: 2 mg/kg reserpine and 100 mg/kg MYP treatment (n = 7). Data are expressed as means ± SEM. One-way ANOVA test with Tukey post-hoc tests executed. $^{*}p < 0.05$, $^{***}p < 0.001$ vs. CON.

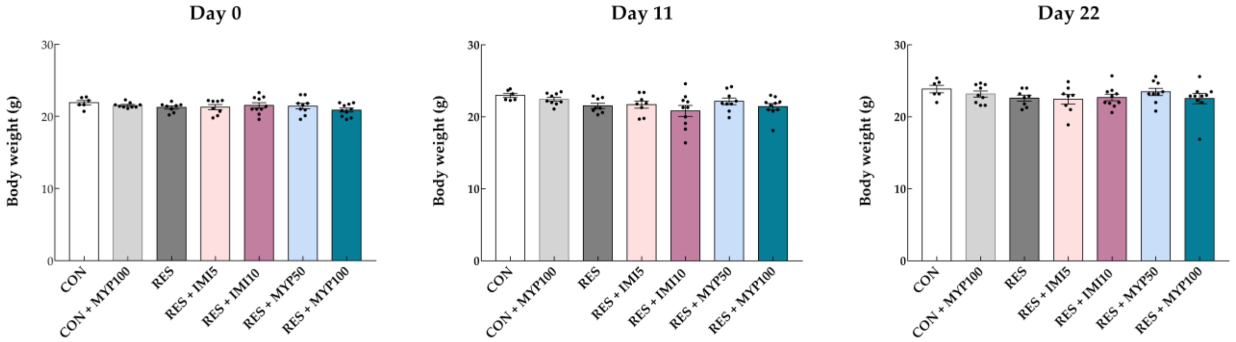

Figure S4. Body weight of all groups on days 0 (before reserpine treatment), 11 (after reserpine treatment), and 22 (after MYP treatment). There was no significant change in the body weight of mice on days 0, 11, and 22 in all groups. CON: control (n = 5), CON + MYP100: 100 mg/kg MYP treatment (n = 9), RES: 2 mg/kg reserpine treatment (n = 7), RES + IMI5: 2 mg/kg reserpine and 5 mg/kg imipramine treatment (n = 8), RES + IMI10: 2 mg/kg reserpine and 10 mg/kg imipramine treatment (n = 10), RES + MYP50: 2 mg/kg reserpine and 50 mg/kg MYP treatment (n = 9), RES + MYP100: 2 mg/kg reserpine and 100 mg/kg MYP treatment (n = 7). Data are expressed as means ± SEM. One-way ANOVA test with Tukey post-hoc tests executed.

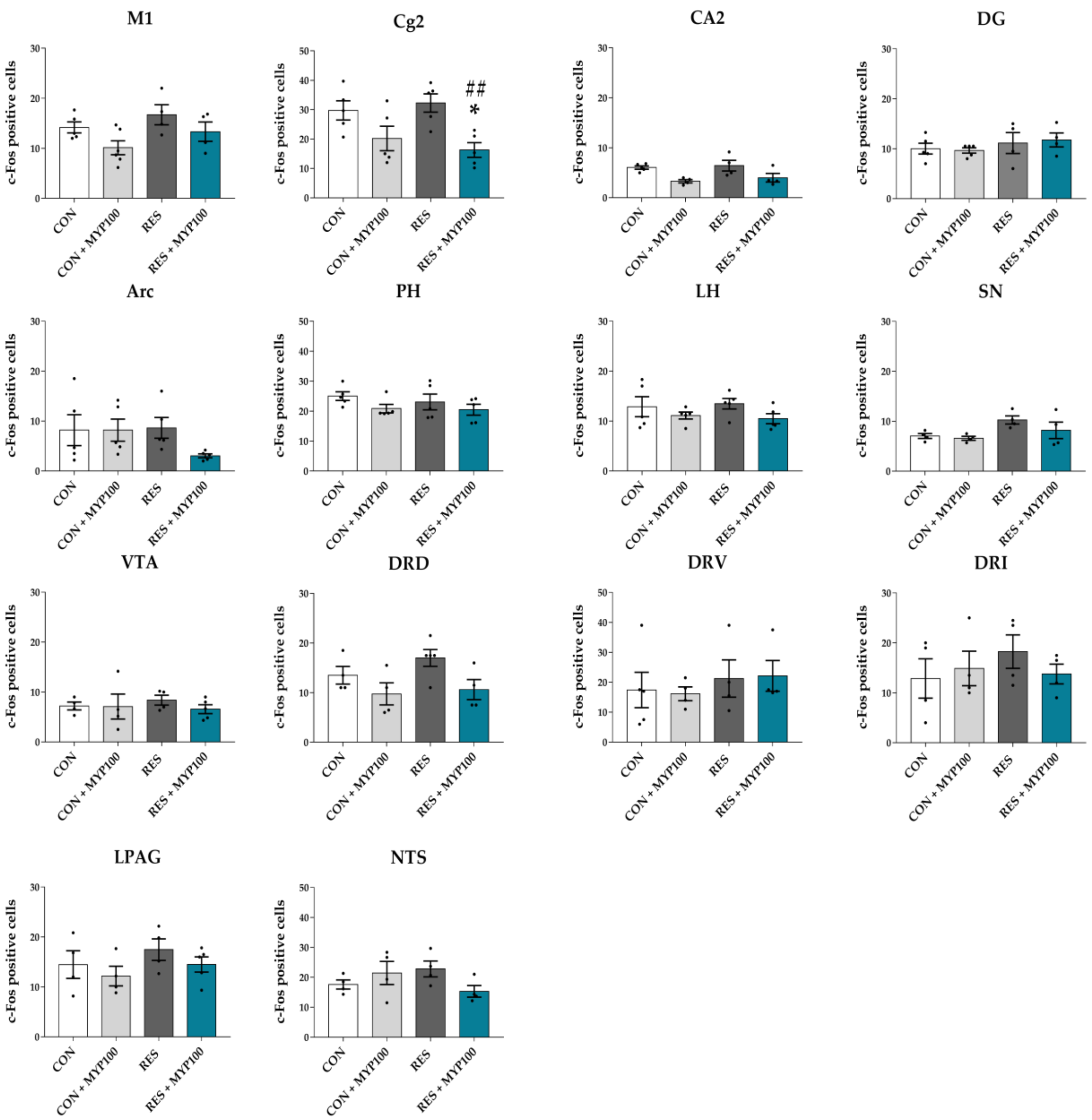

Figure S5. Alteration of c-Fos expression by myelophil (MYP) treatment in each brain regions. The number of c-Fos positive cells in motor cortex area 1 (M1), cingulate area (Cg2), cornu ammonis area 2 (CA2) and dentate gyrus (DG) of hippocampus, arcuate nucleus (Arc), posterior hypothalammic area (PH), lateral hypothalamic area (LH), substantia nigra (SN), ventral tegmental area (VTA), dorsal part of the dorsal raphe nucleus (DRD), ventral part of the dorsal raphe nucleus (DRV), interfascicular part of the dorsal raphe nucleus (DRI), lateral periaqueductal gray (LPAG), and nucleus of solitary tract (NTS) regions were analyzed. CON: control (n = 5), CON + MYP100: 100 mg/kg MYP treatment (n = 5), RES: 2 mg/kg of reserpine treatment (n = 5), RES + MYP100: 2 mg/kg of reserpine followed by 100 mg/kg of MYP treatment (n = 5). Data are expressed as means ± SEM. One-way ANOVA test with Tukey post-hoc tests executed. $^{*}p < 0.05$ vs. CON; $^{\#\#}p < 0.01$ vs. RES.

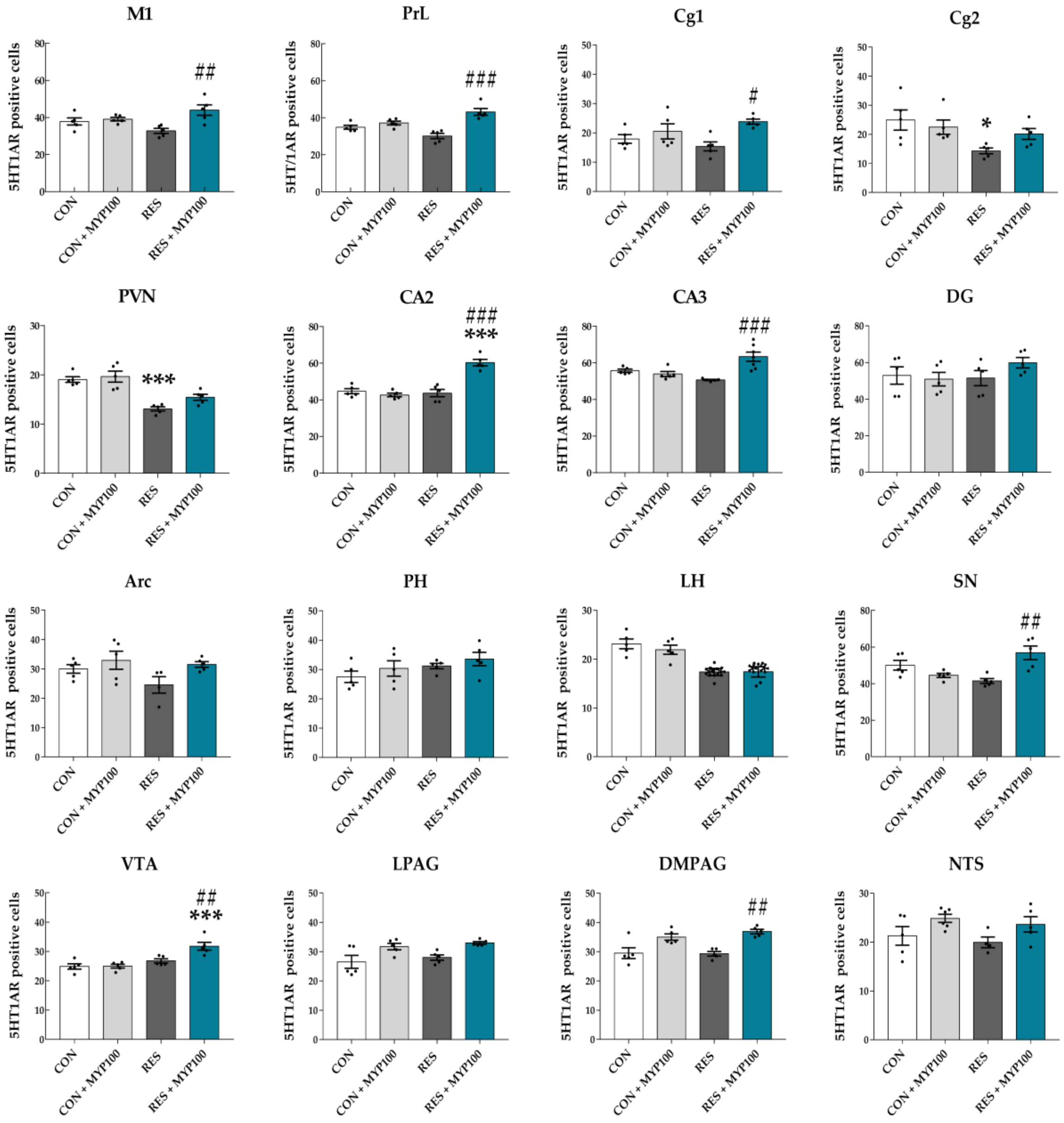

Figure S6. Changes of 5-HT1A receptor (5-HT1AR) expression in each brain region following myelophil (MYP) treatment. The number of 5-HT1AR positive cells in motor cortex area 1 (M1), prelimbic cortex (PrL), cingulate area (Cg) 1, Cg2, paraventricular nucleus (PVN), cornu ammonis area (CA) 2, CA3, and dentate gyrus (DG) of hippocampus, arcuate nucleus (Arc), posterior hypothalamic area (PH), lateral hypothalamic area (LH), substantia nigra (SN), ventral tegmental area (VTA), lateral periaqueductal gray (LPAG), dorsomedial periaqueductal gray (DMPAG), and nucleus of solitary tract (NTS) regions were analyzed. CON: control (n = 5), CON + MYP100: 100 mg/kg MYP treatment (n = 5), RES: 2 mg/kg of reserpine treatment (n = 5), RES + MYP100: 2 mg/kg of reserpine followed by 100 mg/kg of MYP treatment (n = 5). Data are expressed as means ± SEM. One-way ANOVA test with Tukey post-hoc tests executed. $^{*}p < 0.05$, $^{***}p < 0.001$ vs. CON; $^{\#}p < 0.05$, $^{\#\#}p < 0.01$, $^{\#\#\#}p < 0.001$ vs. RES.

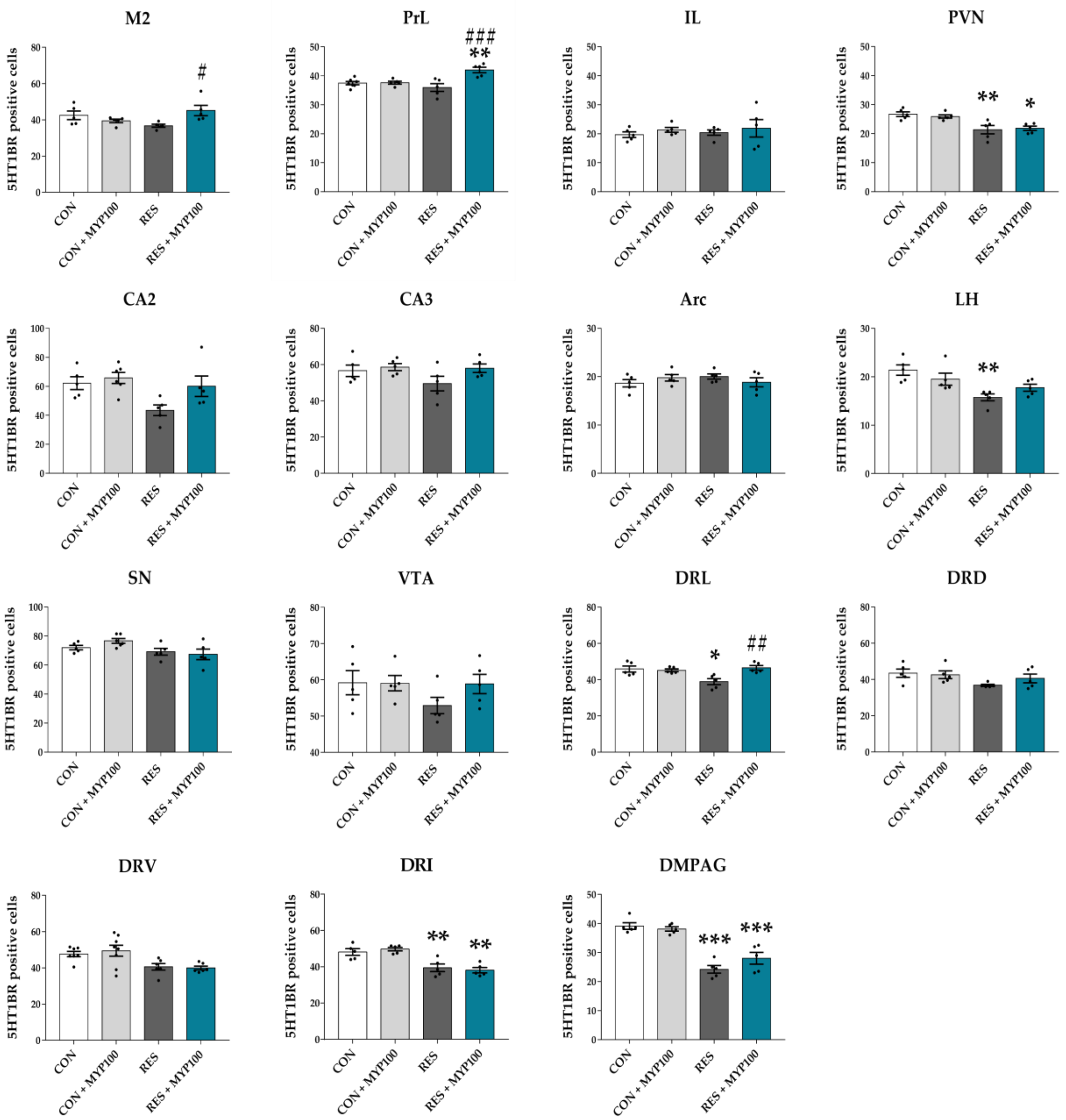

Figure S7. Changes of 5-HT1B receptor (5-HT1BR) expression in each brain region following myelophil (MYP) treatment. The number of 5-HT1BR positive cells in motor cortex area 2 (M2), prelimbic cortex (PrL), infralimbic cortex (IL), paraventricular nucleus (PVN), cornu ammonis area (CA) 2 and CA3 of hippocampus, arcuate nucleus (Arc), lateral hypothalamic area (LH), substantia nigra (SN), ventral tegmental area (VTA), lateral part of the dorsal raphe nucleus (DRL), dorsal part of the dorsal raphe nucleus (DRD), ventral part of the dorsal raphe nucleus (DRV), interfascicular part of the dorsal raphe nucleus (DRI), and dorsomedial periaqueductal gray (DMPAG) regions were analyzed. CON: control (n = 5), CON + MYP100: 100 mg/kg MYP treatment (n = 5), RES: 2 mg/kg of reserpine treatment (n = 5), RES + MYP100: 2 mg/kg of reserpine followed by 100 mg/kg of MYP treatment (n = 5). Data are expressed as means ± SEM. One-way ANOVA test with Tukey post-hoc tests executed. $^{*}p < 0.05$, $^{**}p < 0.01$, $^{***}p < 0.001$ vs. CON; $^{\#}p < 0.05$, $^{\#\#}p < 0.01$, $^{\#\#\#}p < 0.001$ vs. RES.

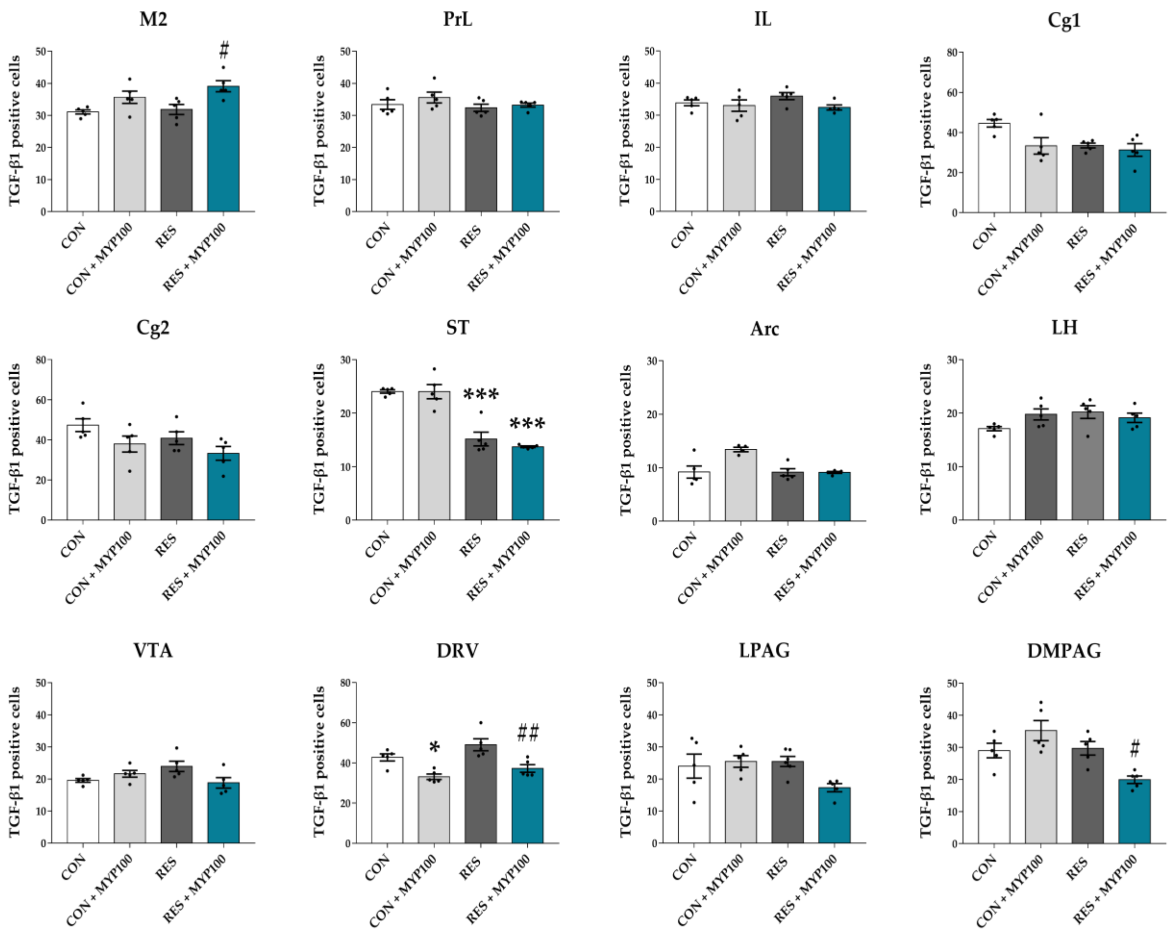

Figure S8. Inhibition of TGF-β1 expression in each brain region by myelophil (MYP) treatment. The number of TGF-β1 positive cells in motor cortex area 2 (M2), prelimbic cortex (PrL), infralimbic cortex (IL), cingulate area (Cg) 1, Cg2, striatum (ST), arcuate nucleus (Arc), Lateral hypothalamic area (LH), ventral tegmental area (VTA), ventral part of the dorsal raphe nucleus (DRV), lateral periaqueductal gray (LPAG), and dorsomedial periaqueductal gray (DPAG) regions were analyzed. CON: control (n = 5), CON + MYP100: 100 mg/kg MYP treatment (n = 5), RES: 2 mg/kg of reserpine treatment (n = 5), RES + MYP100: 2 mg/kg of reserpine followed by 100 mg/kg of MYP treatment (n = 5). Data are expressed as means ± SEM. One-way ANOVA test with Tukey post-hoc tests executed. $^{*}p < 0.05$, $^{***}p < 0.001$ vs. CON; $^{\#}p < 0.05$, $^{\#\#}p < 0.01$ vs. RES.
